# CelltrackR: An R package for fast and flexible analysis of immune cell migration data^☆^

**DOI:** 10.1016/j.immuno.2021.100003

**Published:** 2021-07-31

**Authors:** Inge M.N. Wortel, Annie Y. Liu, Katharina Dannenberg, Jeffrey C. Berry, Mark J. Miller, Johannes Textor

**Affiliations:** a Department of Tumor Immunology, Radboud Institute of Molecular Life Sciences, Geert Grooteplein 26-28, Nijmegen, the Netherlands; b Institute for Theoretical Computer Science, Universitat zu Lubeck, Ratzeburger Allee 160, Lubeck, Germany; c Division of Infectious Diseases, Washington University School of Medicine, 4523 Clayton Avenue, St Louis, USA

**Keywords:** Immune cell migration, Cell track analysis, Two-photon imaging, Modeling, Motion statistics

## Abstract

Visualization of cell migration via time-lapse microscopy has greatly advanced our understanding of the immune system. However, subtle differences in migration dynamics are easily obscured by biases and imaging artifacts. While several analysis methods have been suggested to address these issues, an integrated tool implementing them is currently lacking. Here, we present celltrackR, an R package containing a diverse set of state-of-the-art analysis methods for (immune) cell tracks. CelltrackR supports the complete pipeline for track analysis by providing methods for data management, quality control, extracting and visualizing migration statistics, clustering tracks, and simulating cell migration. CelltrackR supports the analysis of both 2D and 3D cell tracks. CelltrackR is an open-source package released under the GPL-2 license, and is freely available on both GitHub and CRAN. Although the package was designed specifically for immune cell migration data, many of its methods will also be of use in other research areas dealing with moving objects.

## 1. Introduction

The ability to visualize immune cell migration using time-lapse microscopy has allowed researchers to start unraveling the cellular mechanisms underlying immunity, infection, cancer, and chronic inflammation [1], but the new data have also raised many questions. To truly understand how immune cells adjust their migration mode in different contexts, reliable quantification methods are needed.

A major challenge in extracting robust conclusions from immune cell migration data is that differences are often hard to detect, and can be obscured by imaging artifacts and biases in the analysis [2]. Yet even very subtle differences in migration statistics can have large functional consequences on time scales beyond that of the imaging experiment [3,4]. Although novel analysis methods and modeling approaches have been developed to deal with these issues, these are often implemented in custom-made scripts—hampering their widespread use by the community. An accessible tool integrating these different methods is currently lacking.

**Fig. 1.**
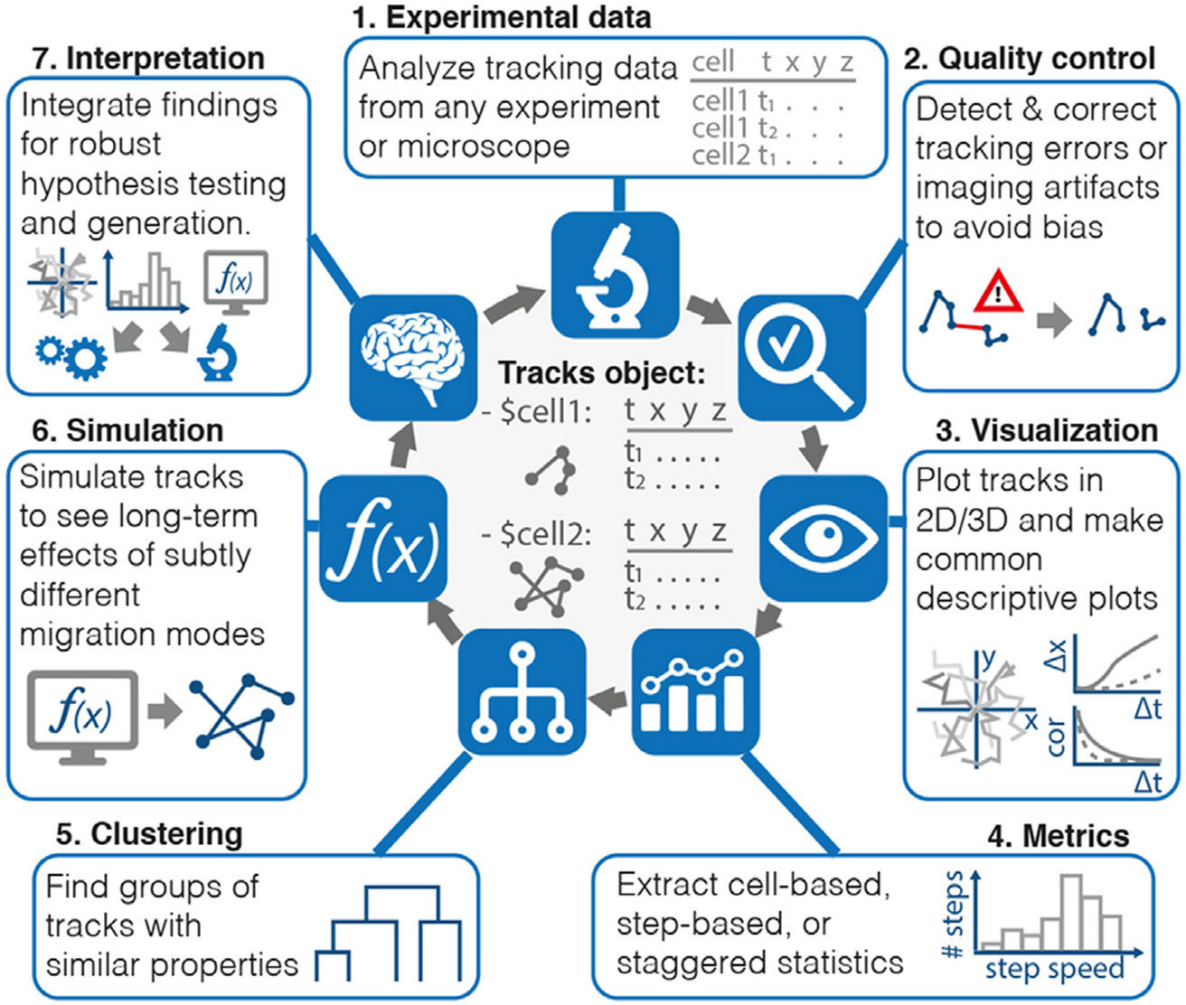
CelltrackR supports the full pipeline from cell migration data to their interpretation. The package implements a new data structure for rapid quantification of migration metrics on track datasets—the *tracks object*—as well as methods for track quality control, quantification, visualization, clustering, and simulation.

We here present celltrackR, an R package for the robust quantification and interpretation of immune cell migration data. Building on the powerful statistical and visualization methods already available within the R programming language [5], celltrackR supports the full workflow from visualizing and quantifying cell tracks to modeling and inferring robust conclusions (Fig. 1).

## 2. Implementation

### 2.1. Tracks and data structures

Cell migration data are typically stored as cell *tracks*, tables linking the position of each cell to the corresponding timepoint in time-lapse images. Thus, celltrackR is compatible with migration data from any experiment or microscope using (variations of) this standardized format (Supplementary file 1).

CelltrackR implements a dedicated data structure, the *tracks object* (Fig. 1). This object is a list with a coordinate matrix for each track in the dataset, which allows rapid computation of different metrics on a dataset. The package also supports conversion between track objects and other data structures (such as the R dataframe) for compatibility with custom analyses and other R packages.

### 2.2. Quality control and preprocessing

CelltrackR contains several methods for detecting tracking errors and preprocessing tracks, including track interpolation, drift correction, and several angle analysis methods [2]; see Supplementary files 2, 3, and 7 for details.

### 2.3. Motility metrics

CelltrackR contains a range of motility statistics designed to characterize cell speed, straightness, and directionality [6,7]. While it is possible to assess these statistics on tracks from individual cells, it has been shown that this “cell-based” method can introduce biases in the analysis [2]. Alternative “step-based” [2], “staggered” [6], and combined [8] approaches therefore compute these metrics on local parts of tracks instead. CelltrackR was designed for compatibility with each of these analysis methods, allowing rapid computation of both existing and custom migration statistics in a cell-based, step-based, or staggered manner (see Supplementary files 4,7).

### 2.4. Customization

Because new track statistics are still developed constantly, the package was designed to be easily extensible by custom track measures—the user only needs to write a function that computes the desired statistic on one single input track. Such functions can be supplied as arguments to many other methods of the package, and be used in the same way as existing methods (see Supplementary files 4,7).

### 2.5. Visualization and statistical analysis

After track quantification, users can compare and visualize migration statistics using R’s standard statistical and visualization tools. Popular visualizations such as rose plots, mean squared displacement (MSD) plots, and autocorrelation plots can all be generated in this fashion and can be compared between different experiments. In addition, celltrackR implements *hotellingsTest* for an unbiased visualization and statistical analysis of subtle directionality in a dataset [3] (see also Supplementary files 4,7).

### 2.6. Clustering

Three methods facilitate detection of groups of tracks with similar migration statistics: celltrackR implements methods for clustering tracks (*clusterTracks*), dimensionality reduction (*trackFeatureMap*), and selecting subsets of tracks with similar values, which can then be compared on some other feature (*selectTracks*) (Supplementary file 5,7).

### 2.7. Simulation

To help users explore long-term effects of migration patterns *in silico*, celltrackR also implements three methods for simulating tracks: *bootstrapTrack* for sampling turning angles and displacements directly from a dataset, *brownianTrack* for simulating simple random walks, and *beaucheminTrack* for a random walk variation designed specifically for T cells; this method also allows for directionally biased motion [9,10].

## 3. Methods

The package contains three datasets of immune cell migration, for which methods are briefly described below.

### 3.1. Neutrophils

Two-photon imaging of neutrophil recruitment was performed as previously published [11-13] with modifications to image the ear. Briefly, LysM-GFP mice were infected with S. aureus on their ear. 1.5–2 h post-infection, the mice were anesthetized using isoflurane and their ear was glued to a custom imaging chamber for in vivo imaging. Fluorescence was excited at 900 nm and GFP signal was collected using 495 and 560 nm emission filters. Timelapse recordings of 31 consecutive 2 *μ*m Z-steps (512x512 pixels, 0.800 microns/pixel, 10f average/z) were acquired at 24 s intervals for 40 time points total to record neutrophil motility proximal to the infected wound. Video rendering and cell tracking were completed using Imaris 9.7.2.

### 3.2. B cells and T cells

Two-photon imaging of B and T cell motility was performed as previously published [14] with modifications to image the lymph nodes. Briefly, a CD11c-YFP mouse was injected retro-orbitally with 3 M cells each (50 *μ*L each) of B (GFP) and T (RFP) cells. The next day, the mouse was anesthetized using isoflurane and its lymph nodes were explanted then placed in a perfusion chamber containing DMEM and set to 37 °C for imaging. Fluorescence was excited at 890 nm to collect YFP, RFP, and GFP signal using 480, 526, and 560 nm emission filters. Time-lapse recordings and cell tracks were then obtained as described for neutrophils above.

## 4. Example usage

**Fig. 2.**
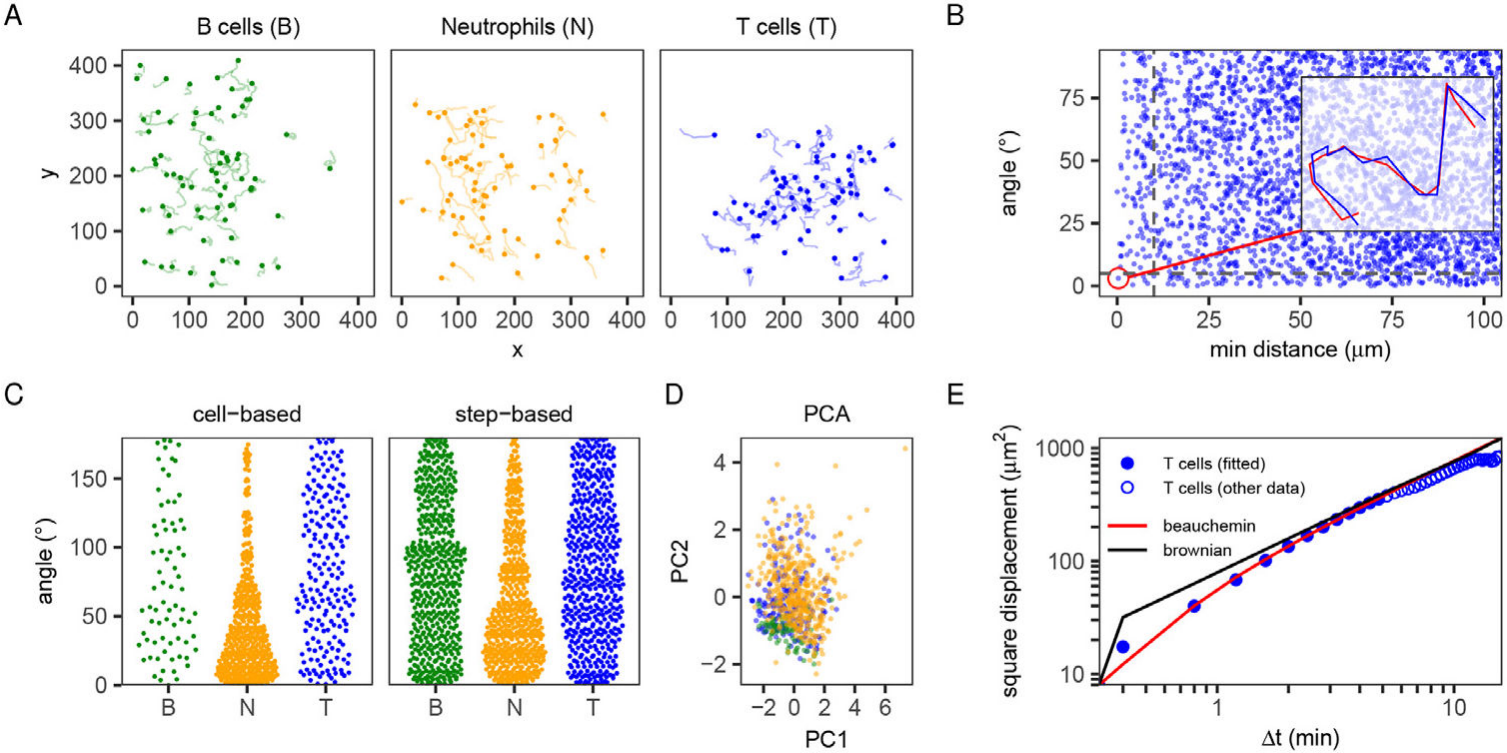
Example analysis of migration data of different immune cells. B cells and T cells were imaged in the cervical lymph node of a healthy mouse, neutrophils proximal to an S. aureus infection in the mouse ear. Data were preprocessed as described in Supplementary file 3; for full code, see Supplementary file 8. (A) Trajectories of the different datasets (for this example, 70 tracks per cell type were sampled randomly to avoid cluttered plots for larger data sets). (B) Angle analysis reveals a (simulated) double tracking error in the T-cell dataset. Each point is one pair of tracks, plotted as the minimum distance between them (at any point in time) versus the angle between their overall displacement vectors (from the beginning to the end of each track). The doubly tracked cell appears at low distance and angle; the corresponding track and its duplicate are shown in the inset (see also Supplementary file 2). (C) Cell-based and step-based angles to direction (1, −1) for the T cell (T), B cell (B), and neutrophil (N) datasets (see Supplementary file 4 for details). (D) Principal Component Analysis (PCA) based on multiple features (speed, mean turning angle, squared displacement, and outreach ratio; see Supplementary files 5 and 7) reveals which tracks have similar motility. Each point represents a track, and colors represent the dataset. (E) Comparison of the MSD of the T-cell dataset (blue circles) versus 2 fitted models (brownian motion and the Beauchemin model [9]). Only the solid blue points were used to fit on, since the empirical MSD at longer timescales is affected by the limited imaging window (an artifact); see Supplementary file 6 for details on fitting models.

The celltrackR package contains three example datasets showing the motility of B cells, T cells, and neutrophils in different settings: B and T cells were imaged in the cervical lymph node of healthy mice, and neutrophils were imaged proximal to an S. aureus infection in the ear (see methods for details). Motility in these scenarios is well-known to be different; neutrophils responding to an infection migrate in a directed fashion, whereas B and T cells in the lymph node migrate in patterns resembling a random walk. We here present an example analysis comparing these motility patterns to illustrate how celltrackR supports the cell track analysis workflow (Fig. 2).

### 4.1. Loading data from different experimental set-ups

A typical analysis workflow starts with reading in trajectories from a text file (see Supplementary files 1, 7 for details), after which they can be further analyzed using the package. For a visual inspection of the T-cell, B-cell, and neutrophil datasets, trajectories were plotted for each (Fig. 2A; for simplicity, tracks were projected on the xy-plane from here on, but 3D analysis works in the same way). While B-cell and T-cell tracks were oriented in various directions, neutrophil motility seemed to be biased in the (1, −1) direction. Thus, plotting trajectories may provide a first (qualitative) indication of differences between datasets.

### 4.2. Quality control and preprocessing

Before moving on to further, quantitative analyses on a set of tracks, it is important to check for (and correct) any errors in the data. To demonstrate how celltrackR can help identify errors, we simulated a “double tracking” error by duplicating one of the T-cell trajectories (adding some Gaussian noise to each coordinate, simulating a case where a cell was accidentally tracked twice). Such duplicate tracks are easily detected in a distance-angle plot of all track pairs (Fig. 2B). For details and other quality control and correction methods, see Supplementary files 2, 3, and 7.

### 4.3. Extracting and visualizing cell- or step-based statistics

To quantify the directional bias observed in neutrophil trajectories (Fig. 2A) and compare this to the other datasets, the angle to the (1, −1) direction was computed in both a cell-based and step-based manner (Fig. 2C). While T-cell and B-cell angles followed roughly uniform distributions, neutrophil angles were biased towards low angles. Thus, these results confirm quantitatively that motion in the neutrophil dataset is more biased compared to that in the T-cell and B-cell datasets.

### 4.4. Grouping and clustering tracks

To demonstrate how dimensionality reduction can contribute to motility analysis, we pooled all datasets and performed a principal component analysis (PCA), using (cell-based) speed, mean turning angle, squared displacement, and outreach ratio as input features. While neutrophils were nicely separated from B cells, T cells did not form a separate cluster, with some resembling B cells while other resembled neutrophils (Fig. 2D). This analysis shows that even when underlying motility modes are different, individual tracks can still be similar between different datasets. For details and alternative dimensionality reduction and clustering methods, see Supplementary files 5,7.

### 4.5. Simulating immune cell migration

Since migration experiments typically measure immune cell movement over short timeframes, it can be difficult to estimate whether a subtle difference in migration statistics has any functional significance. Simulation can be a powerful tool to extrapolate experimentally observed changes in migration to their functional effects in the long run [3,4,9].

To illustrate how such models can be fitted to a dataset, we fitted the MSD curve of the T-cell dataset using two different models: simple Brownian motion, and the Beauchemin model developed specifically for lymphocyte migration [9,10] (for details on the fitting procedure, see Supplementary files 6,8). As expected, on short time scales, the T-cell MSD curve was well-described by the Beauchemin model, but not by Brownian motion (Fig. 2E).

## 5. Discussion

### 5.1. Comparison to available software

While several tools exist for the analysis of cell migration from time-lapse imaging data, most of these tools focus on the computer vision problem of extracting tracks from the supplied images and provide only some basic statistical analysis (such as speed) on the tracks produced. In addition, many tools only support track analysis in 2D and cannot handle 3D tracks from two-photon imaging experiments commonly performed by immunologists (Table 1). CelltrackR was therefore designed specifically for the analysis of both 2D and 3D tracks.

Two other tools exist that focus on track analysis rather than track generation: CellMissy [15] and the Ibidi Chemotaxis & Migration tool [16]—of which only the latter supports 3D data (Table 1). However, both of these are aimed at users that wish to analyze data produced by standard migration assays. They are built with a graphical user interface (GUI) that makes it easy to perform these standard analyses, but is not well-suited for performing custom, in-depth, exploratory analyses on a complex dataset. Furthermore, these tools lack most of the quality control and angle analysis methods that have proven extremely powerful for interpretating immune cell migration data (see [2]).

CelltrackR therefore implements a wide array of methods commonly used in the immune cell migration field. Furthermore, it extends these with methods for dimensionality reduction, clustering, and simulation to support inference from migration data sets (Table 1). The package is aimed at data scientists who wish to perform an in-depth exploration of cell migration data using both existing and custom analysis methods—supporting the development of novel methodologies. For this reason, celltrackR is implemented not as a GUI with predefined methods, but as an R package that can easily be integrated with other data science tools available in the R language.

### 5.2. Further notes and future development

CelltrackR has been designed as part of the MotilityLab project [26], which is still under development. Indeed, an earlier version of the celltrackR package was called “MotilityLab”. The current version of celltrackR is an extension of this package, released under a new name to make the package easier to find.

While the R package itself is designed for programmers that wish to explore migration data in a flexible manner, the MotilityLab website provides a simple GUI frontend to several popular functions in the package [26]. More celltrackR functions will be made available from this GUI in the future.

## 6. Conclusion

CelltrackR is the first R package designed specifically for the in-depth analysis of (immune) cell migration data in both 2D and 3D. The implementation in the R programming language allows users easy access to existing analysis methods consolidated by the immune cell migration field, while still also providing the flexibility needed for custom analyses. Together with the many resources for data visualization and statistical analysis already available in the R language, this makes celltrackR a powerful tool for exploring and analyzing cell migration data. Specifically, the package implements a data structure for migration data that allows rapid computation of a diverse array of statistics on immune cell tracks, is compatible with both cell- and step-based approaches from literature, and can easily be extended with future methods if required. It also supports several important quality controls suggested in literature and allows users to combine track analysis, visualization, clustering, and simulation in a single platform.

## Supplementary Material

Supplemental movies and figures

## Figures and Tables

**Table 1:** Comparison between celltrackR and existing software for cell migration data. The table lists accessibility and implemented functionalities for each available resource (as assessed from the online documentation provided by the software authors).[17-25]

Name	Description				Tracking, QC, preprocessing									Analysis scales					Analysis metrics											Angles/directions						Visualization				Inference			
	Format	Free (including dependencies)	Open Source	Support for 3D tracking	Track from image	Load tracks from other software	Detect gaps	Detect/filter short tracks	Detect/correct drift	Detect tracking errors	Interpolate tracks	Automated filtering	Select subtracks	Instantaneous metrics	Cell-based metrics	Step-based metrics	Staggered metrics	Population metrics	Displacement	Max displacement	Speed	Turning angles	Turning dot product	Track length	Straightness	Asphericity	Outreach ratio	Displacement ratio	Interface for custom metric	Angle/distance to point	Angle to direction	Angle/distance to plane	Forward migration index (FMI)	Angles between (sub)tracks	Test lor directionality	Track visualization	MSD plot	Autocorrelation plot	Plot metrics and/or angles	Feature dimensionality reduction	Track clustering	Statistical testing on track metrics	Track simulation
celltrackR	RP	+	+	+	−	+	A	+	TB	A	+	+	A	+	+	+	+	+	+	+	+	+	+	+	+	+	+	+	+	+	+	+	−	+	H	+	+	+	C	+	+	+*	+
CellMissy [15]	JG	+	+	−	−	+	−	−	−	−	+	+*	−	+	+	+	−	+	+	+	+	+	−	+	−	−	+	+	−	−	−	−	−	−	−	+	+	+	PB	−	−	+	−
Chemotaxis & Migration [16]	IP,S	+	+	+	−	+	−	−	−	−	−	+*	−	+	+	−	−	+	+	−	+	−	−	+	+	−	−	−	−	−	−	−	+	−	R	+	−	−	PB	−	−	−	−
Cellprofiler Tracer [17]	JG	+	+	−	+	−	A	+	−	A	−	−	M	+	−	−	−	−	+	−	−	−	−	−	−	−	−	−	−	−	−	−	−	−	−	+	−	−	−	−	−	−	−
Icy* [18]	IP	+	+	+	+	−	−	−	−	−	−	+	−	+	+	−	−	+	+	+	+	−	−	+	−	−	−	−	−	−	−	−	−	−	−	+	+	−	PB	−	−	−	−
TrackMate [19]	IP	+	+	+	+	−	−	+	−	−	−	+	−	+	+	−	−	+	+	+	+	−	−	+	+	−	−	−	+	−	−	−	+	−	−	+	−	−	PB	−	−	−	−
iTrack4U [20]	JG	+	+	−	+	−	−	−	−	−	−	−	−	+	+	−	−	+	+	+	+	+	−	+	+	−	−	−	−	−	s	−	−	−	−	+	−	−	−	−	−	−	−
CellTrack [21]	S	+	+	−	+	−	−	−	−	−	−	−	−	+	+	−	−	−	−	−	+	−	−	+	−	−	−	−	−	−	−	−	−	−	−	+	−	−	PB	−	−	−	−
CellTracker [22]	MP	−	+	−	+	−	M	+	IB	M	+	−	−	+	+	−	−	+	+	+	+	+	−	+	−	−	+	−	−	s	−	−	−	−	−	+	−	−	−	−	−	−	−
ImariesTrack [23]	S	−	−	+	+	−	M	+	IB	−	−	+	−	+	+	−	−	+	+	−	+	−	−	+	+	−	−	−	−	−	−	−	−	+	−	+	−	−	PB	−	−	−	−
MetaMorph [24]	S	−	−	−	+	−	−	−	−	−	−	−	−	+	+	−	−	+	+	−	+	−	−	−	−	−	−	−	−	−	−	−	−	−	−	+	−	−	PB	−	−	−	−
Velocity [25]	S	−	−	+	+	−	−	−	−	−	−	−	−	+	+	−	−	+	−	−	+	−	−	+	−	−	−	−	−	−	−	−	−	−	−	+	−	−	PB	−	−	−	−
*With PACT, Track Manager, and Motion Profiler plugins	R package (RP), Java GUI (JG), ImageJ/Icy plugins (IP), Standalone (S), Matlab plugin (MP)				automated/manual (A/M) track/image-based (TB/IB), *only based on distance/velocity																									only with respect to specific point/direction (s), Hotelling's test (H), Rayleigh Test (R)						pre-built plots only (PB), custom plots of any metric (C)				*Using any statistical testing options from the R language			
