# Supplementary material for: CelltrackR: An R package for fast and flexible analysis of immune cell migration data
Source: Immunoinformatics (Amst). Author manuscript; Available in PMC 2023 Apr 6. (PMC10079262; doi:10.1016/j.immuno.2021.100003)
Supplement: Supplemental movies and figures [file NIHMS1884788-supplement-Supplemental_movies_and_figures.zip › SF1-reading-converting-data.html]

Reading, Converting, and Filtering Tracking Data


# Reading, Converting, and Filtering Tracking Data

#### Inge Wortel

#### 2021-07-07

# Introduction

The package implements a special data structure, the tracks object, to allow rapid computation of different analysis metrics on cell tracks. This tutorial will show how to load tracking data, how to deal with tracking objects, how to filter and subset data, and how to convert between track objects and other datastructures.

# 1 Reading in data

First load the package:

```
library( celltrackR )
library( ggplot2 )
```

### 1.1 Input data format

Tracking data is usually stored as a table, with columns indicating the cellid, time, and coordinates of each measured point. Here we have an example in the file “t-cells.txt”, which we can read in as a normal dataframe:

```
d <- read.table( system.file("extdata", "t-cells.txt", package="celltrackR" ) )
str(d)
```

```
## 'data.frame':    5157 obs. of  6 variables:
##  $ V1: int  2 3 4 5 6 7 8 9 10 11 ...
##  $ V2: int  1 1 1 1 1 1 1 1 1 1 ...
##  $ V3: int  48 72 96 120 144 168 192 216 240 264 ...
##  $ V4: num  90.9 89.6 88.7 87.3 86.3 ...
##  $ V5: num  65.4 64.9 67.1 68.2 67.9 ...
##  $ V6: num  -6417 -6420 -6422 -6424 -6425 ...
```

```
head(d)
```

```
##   V1 V2  V3      V4      V5       V6
## 1  2  1  48 90.8534 65.3943 -6416.80
## 2  3  1  72 89.5923 64.9042 -6419.93
## 3  4  1  96 88.6958 67.1125 -6421.80
## 4  5  1 120 87.3437 68.2392 -6424.08
## 5  6  1 144 86.2740 67.9236 -6425.14
## 6  7  1 168 84.0549 68.2502 -6426.68
```

The result is a normal dataframe, where here we have an index for the time point in the first column, cell id in the second column, the actual time (in seconds) in the third column, and \((x,y,z)\) coordinates in columns 4:6.

# 1.2 Directly reading in data as a tracks object

While we can read tracks as a dataframe by using R’s basic function `read.table()`, the function `read.tracks.csv()` allows to read in data directly as a *tracks object*, a special data structure designed for efficient handling of tracking data.

Applying this to the same file as before:

```
t <- read.tracks.csv( system.file("extdata", "t-cells.txt", package="celltrackR" ), 
              header = FALSE, 
                      id.column = 2, time.column = 3, pos.columns = 4:6 )
plot(t)
```

where we have to specify `header=FALSE` because the file does not contain any column headers. Note that `read.tracks.csv()` also works with non-csv text files, as long as the data is organised with separate columns for track id, time index, and coordinates. See the documentation at `?read.tracks.csv` for details.

These tracks are of T cells imaged in the cervical lymph node of a healthy mouse; they are the raw data from which the `TCells` dataset in the package was obtained. See the vignette on preprocessing the package datasets for details.

# 2 The tracks object

### 2.1 The tracks object data structure

The *tracks object* is a special datastructure that allows efficient handling of track datasets. As an example, we will use the tracks loaded in the previous section.

A tracks object has the form of a list, where each element of the list is a track of a single cell:

```
# Structure of the TCells object
str( t, list.len = 3 )
```

```
## List of 258
##  $ 1   : num [1:39, 1:4] 48 72 96 120 144 168 192 216 240 264 ...
##   ..- attr(*, "dimnames")=List of 2
##   .. ..$ : NULL
##   .. ..$ : chr [1:4] "t" "x" "y" "z"
##  $ 2   : num [1:7, 1:4] 48 72 96 120 144 ...
##   ..- attr(*, "dimnames")=List of 2
##   .. ..$ : NULL
##   .. ..$ : chr [1:4] "t" "x" "y" "z"
##  $ 3   : num [1:19, 1:4] 24 48 72 96 120 144 168 192 216 240 ...
##   ..- attr(*, "dimnames")=List of 2
##   .. ..$ : NULL
##   .. ..$ : chr [1:4] "t" "x" "y" "z"
##   [list output truncated]
##  - attr(*, "class")= chr "tracks"
```

```
# This object is both a list and a "tracks" object
is.list( t )
```

```
## [1] TRUE
```

```
is.tracks( t )
```

```
## [1] TRUE
```

```
# The first element is the track of the first cell in the data:
head( t[[1]] )
```

```
##        t       x       y        z
## [1,]  48 90.8534 65.3943 -6416.80
## [2,]  72 89.5923 64.9042 -6419.93
## [3,]  96 88.6958 67.1125 -6421.80
## [4,] 120 87.3437 68.2392 -6424.08
## [5,] 144 86.2740 67.9236 -6425.14
## [6,] 168 84.0549 68.2502 -6426.68
```

Each track in the tracks object is a matrix with coordinates at different timepoints for each cell. The cell id is no longer a column in this matrix, as tracks belonging to different cells are stored in different elements of the tracks object list.

### 2.2 Subsetting data

Note that we can subset the track matrix of an individual track using the double square brackets:

```
# Get the first track
t1 <- t[[1]]
str(t1)
```

```
##  num [1:39, 1:4] 48 72 96 120 144 168 192 216 240 264 ...
##  - attr(*, "dimnames")=List of 2
##   ..$ : NULL
##   ..$ : chr [1:4] "t" "x" "y" "z"
```

```
# This is no longer a tracks object, but a matrix
is.tracks( t1 )
```

```
## [1] FALSE
```

```
is.matrix( t1 )
```

```
## [1] TRUE
```

If we now want to plot this track, the plotting method for tracks will not work because this is not recognized as a tracks object. We can use the frunction `wrapTrack()` to “pack” this matrix back into a tracks object:

```
par( mfrow=c(1,2) )
plot( t1, main = "Plotting matrix directly" )
plot( wrapTrack( t1 ), main = "After using wrapTrack()" )
```

Note that we can also achieve this by subsetting with single instead of double brackets:

```
# Get the first track
t1b <- t[1]
str(t1b)
```

```
## List of 1
##  $ 1: num [1:39, 1:4] 48 72 96 120 144 168 192 216 240 264 ...
##   ..- attr(*, "dimnames")=List of 2
##   .. ..$ : NULL
##   .. ..$ : chr [1:4] "t" "x" "y" "z"
##  - attr(*, "class")= chr "tracks"
```

```
# This remains a track object
is.tracks( t1b )
```

```
## [1] TRUE
```

In the same way, we can also subset multiple tracks at once

```
# Get the first and the third track
t13 <- t[c(1,3)]
str(t13)
```

```
## List of 2
##  $ 1: num [1:39, 1:4] 48 72 96 120 144 168 192 216 240 264 ...
##   ..- attr(*, "dimnames")=List of 2
##   .. ..$ : NULL
##   .. ..$ : chr [1:4] "t" "x" "y" "z"
##  $ 3: num [1:19, 1:4] 24 48 72 96 120 144 168 192 216 240 ...
##   ..- attr(*, "dimnames")=List of 2
##   .. ..$ : NULL
##   .. ..$ : chr [1:4] "t" "x" "y" "z"
##  - attr(*, "class")= chr "tracks"
```

Note that the track ids are strings that do not always correspond to the index of the track in the dataset. If we want the ones with ids 1 and 3, we can subset using the track name as a character string:

```
# Get tracks with ids 1 and 3
t13b <- t[c("1","3")]
str(t13b)
```

```
## List of 2
##  $ 1: num [1:39, 1:4] 48 72 96 120 144 168 192 216 240 264 ...
##   ..- attr(*, "dimnames")=List of 2
##   .. ..$ : NULL
##   .. ..$ : chr [1:4] "t" "x" "y" "z"
##  $ 3: num [1:19, 1:4] 24 48 72 96 120 144 168 192 216 240 ...
##   ..- attr(*, "dimnames")=List of 2
##   .. ..$ : NULL
##   .. ..$ : chr [1:4] "t" "x" "y" "z"
##  - attr(*, "class")= chr "tracks"
```

### 2.3 Using tracks objects in combination with R’s lapply and sapply

Because tracks objects are lists, we can make use of R’s `lapply()` and `sapply()` functions to compute metrics or manipulate tracks efficiently.

For example, if we want to compute the speed of each track, we simply use:

```
speeds <- sapply( t, speed )
head(speeds)
```

```
##          1          2          3          4          5          6 
## 0.18035686 0.05231612 0.09511618 0.14386174 0.20168555 0.11651339
```

Note that `sapply()` applies the `speed()` function to each *matrix* in the track list (analogous to subsetting with double brackets). Thus, the `speed()` function sees an individual track matrix, not a tracks object.

Or we can use `lapply()` to manipulate each track in the dataset with some custom function, keeping separate tracks as separate list elements. For example, suppose we wish to remove all data after a given timepoint:

```
# Function to remove all data after given timepoint
# x must be a single track matrix, which is what this function will
# receive from lapply
removeAfterT <- function( x, time.cutoff ){
  
  # Filter out later timepoints
  x2 <- x[ x[,"t"] <= time.cutoff, ]
  
  # Return the new matrix, or NULL if there are no timepoints before the cutoff
  if( nrow(x2) == 0 ){
    return(NULL)
  } else {
    return(x2)
  }
}

# Call function on each track using lapply
filtered.t <- lapply( t, function(x) removeAfterT( x, 200 ) )

# Remove any tracks where NULL was returned
filtered.t <- filtered.t[ !sapply( filtered.t, is.null )]
```

Note that `lapply()` returns list but not a tracks object:

```
str(filtered.t, list.len = 3 )
```

```
## List of 144
##  $ 1   : num [1:7, 1:4] 48 72 96 120 144 ...
##   ..- attr(*, "dimnames")=List of 2
##   .. ..$ : NULL
##   .. ..$ : chr [1:4] "t" "x" "y" "z"
##  $ 2   : num [1:7, 1:4] 48 72 96 120 144 ...
##   ..- attr(*, "dimnames")=List of 2
##   .. ..$ : NULL
##   .. ..$ : chr [1:4] "t" "x" "y" "z"
##  $ 3   : num [1:8, 1:4] 24 48 72 96 120 ...
##   ..- attr(*, "dimnames")=List of 2
##   .. ..$ : NULL
##   .. ..$ : chr [1:4] "t" "x" "y" "z"
##   [list output truncated]
```

```
is.list( filtered.t )
```

```
## [1] TRUE
```

```
is.tracks( filtered.t )
```

```
## [1] FALSE
```

We can fix this by calling `as.tracks()`.

```
filtered.t <- as.tracks( filtered.t )
is.tracks( filtered.t )
```

```
## [1] TRUE
```

```
str(filtered.t, list.len = 1)
```

```
## List of 144
##  $ 1   : num [1:7, 1:4] 48 72 96 120 144 ...
##   ..- attr(*, "dimnames")=List of 2
##   .. ..$ : NULL
##   .. .. [list output truncated]
##   [list output truncated]
##  - attr(*, "class")= chr "tracks"
```

We now have a new tracks object, which contains only tracks that had coordinates at \(t<200\).

### 2.4 Built-in filtering/subsetting functions

The package contains several built-in functions to filter and subset tracks.

The function `filterTracks()` can be used to select tracks with a certain property. For example, to select all tracks with at least 15 steps (16 datapoints):

```
# The filtering function must return TRUE or FALSE for each track given to it
my.filter <- function(x){
  return( nrow(x) > 15 )
}

# Filter with this function using filterTracks
long.tracks <- filterTracks( my.filter, t )

# check the minimum track length; # steps = number of coordinates minus 1
min( sapply( long.tracks, nrow ) - 1 )
```

```
## [1] 15
```

The function `selectTracks()` selects tracks based on upper and lower bounds of a certain measure. For example, we can get the fastest half of the T cells:

```
# Filter with this function using filterTracks
median.speed <- median( sapply( t, speed ) )
fast.tracks <- selectTracks( t, speed, median.speed, Inf )

# these should have a higher mean speed
c( "all tracks" = mean( sapply( t, speed ) ),
   "fastest half" = mean( sapply( fast.tracks, speed ) ) )
```

```
##   all tracks fastest half 
##    0.1650678    0.2255908
```

Another option is to filter not tracks, but timepoints within those tracks. Using the function `subsample()`, we can adjust the time resolution of the data by keeping e.g. only every \(k^{th}\) timepoint:

```
# Lower resolution
lower.res <- subsample( t, k = 2 )

# Plot the result; plot just one track to see the result more clearly
par(mfrow=c(1,2))
plot( t[1], main = "Original data")
plot( lower.res[1], main = "Lower resolution" )
```

### 2.5 Extracting subtracks

The package also contains functions to extract parts of tracks. For example, use `subtracks()` to extract subtracks of a given length:

```
subtrack.nsteps <- 2
t.2steps <- subtracks( t, subtrack.nsteps )
str( t.2steps, list.len = 3 )
```

```
## List of 4641
##  $ 1.1    : num [1:3, 1:4] 48 72 96 90.9 89.6 ...
##   ..- attr(*, "dimnames")=List of 2
##   .. ..$ : NULL
##   .. ..$ : chr [1:4] "t" "x" "y" "z"
##  $ 1.2    : num [1:3, 1:4] 72 96 120 89.6 88.7 ...
##   ..- attr(*, "dimnames")=List of 2
##   .. ..$ : NULL
##   .. ..$ : chr [1:4] "t" "x" "y" "z"
##  $ 1.3    : num [1:3, 1:4] 96 120 144 88.7 87.3 ...
##   ..- attr(*, "dimnames")=List of 2
##   .. ..$ : NULL
##   .. ..$ : chr [1:4] "t" "x" "y" "z"
##   [list output truncated]
##  - attr(*, "class")= chr "tracks"
```

Note that these subtracks overlap:

```
# Last step of the first subtrack and first step of the second are equal
t.2steps[c(1,2)]
```

```
## $`1.1`
##       t       x       y        z
## [1,] 48 90.8534 65.3943 -6416.80
## [2,] 72 89.5923 64.9042 -6419.93
## [3,] 96 88.6958 67.1125 -6421.80
## 
## $`1.2`
##        t       x       y        z
## [1,]  72 89.5923 64.9042 -6419.93
## [2,]  96 88.6958 67.1125 -6421.80
## [3,] 120 87.3437 68.2392 -6424.08
## 
## attr(,"class")
## [1] "tracks"
```

We can prevent this by adjusting the `overlap` argument to 0, or even to negative values so that space is left between the subtracks:

```
t.2steps.b <- subtracks( t, subtrack.nsteps, overlap = 0 )

# No longer any overlap
t.2steps.b[c(1,2)]
```

```
## $`1.1`
##       t       x       y        z
## [1,] 48 90.8534 65.3943 -6416.80
## [2,] 72 89.5923 64.9042 -6419.93
## [3,] 96 88.6958 67.1125 -6421.80
## 
## $`1.3`
##        t       x       y        z
## [1,]  96 88.6958 67.1125 -6421.80
## [2,] 120 87.3437 68.2392 -6424.08
## [3,] 144 86.2740 67.9236 -6425.14
## 
## attr(,"class")
## [1] "tracks"
```

An alternative to `subtracks()` is `prefixes()`, which returns only the first subtrack of a given length from each track:

```
t.prefixes <- prefixes( t, subtrack.nsteps )

# these subtracks come from different cells
t.prefixes[c(1,2)]
```

```
## $`1`
##       t       x       y        z
## [1,] 48 90.8534 65.3943 -6416.80
## [2,] 72 89.5923 64.9042 -6419.93
## [3,] 96 88.6958 67.1125 -6421.80
## 
## $`2`
##       t       x       y        z
## [1,] 48 200.672 38.0959 -6441.29
## [2,] 72 200.464 38.0264 -6441.29
## [3,] 96 200.928 37.7967 -6441.29
```

If we want to extract subtracks starting at a specific timepoint, use `subtracksByTime()`:

```
# Check which timepoints occur in the dataset
tp <- timePoints(t)
tp
```

```
##  [1]  48  72  96 120 144 168 192 216 240 264 288 312 336 360 384 408 432 456 480
## [20] 504 528 552 576 600 624 648 672 696 720 744 768 792 816 840 864 888 912 936
## [39] 960  24
```

```
# Extract all subtracks starting from the third timepoint
t.sbytime <- subtracksByTime( t, tp[3], subtrack.nsteps )

t.sbytime[c(1,2)]
```

```
## $`1`
##        t       x       y        z
## [1,]  96 88.6958 67.1125 -6421.80
## [2,] 120 87.3437 68.2392 -6424.08
## [3,] 144 86.2740 67.9236 -6425.14
## 
## $`2`
##        t       x       y        z
## [1,]  96 200.928 37.7967 -6441.29
## [2,] 120 198.919 38.5586 -6441.29
## [3,] 144 199.684 39.1614 -6441.29
## 
## attr(,"class")
## [1] "tracks"
```

# 3 Converting between tracks objects and other data structures

We can convert between tracks, regular R lists, and dataframes using `as.tracks()`, `as.list()`, or `as.data.frame()`:

```
# Original tracks object
str( t, list.len = 3 )
```

```
## List of 258
##  $ 1   : num [1:39, 1:4] 48 72 96 120 144 168 192 216 240 264 ...
##   ..- attr(*, "dimnames")=List of 2
##   .. ..$ : NULL
##   .. ..$ : chr [1:4] "t" "x" "y" "z"
##  $ 2   : num [1:7, 1:4] 48 72 96 120 144 ...
##   ..- attr(*, "dimnames")=List of 2
##   .. ..$ : NULL
##   .. ..$ : chr [1:4] "t" "x" "y" "z"
##  $ 3   : num [1:19, 1:4] 24 48 72 96 120 144 168 192 216 240 ...
##   ..- attr(*, "dimnames")=List of 2
##   .. ..$ : NULL
##   .. ..$ : chr [1:4] "t" "x" "y" "z"
##   [list output truncated]
##  - attr(*, "class")= chr "tracks"
```

```
# Converted to dataframe
t.df <- as.data.frame(t)
str( t.df )
```

```
## 'data.frame':    5157 obs. of  5 variables:
##  $ id: Factor w/ 258 levels "1","10","100",..: 1 1 1 1 1 1 1 1 1 1 ...
##  $ t : num  48 72 96 120 144 168 192 216 240 264 ...
##  $ x : num  90.9 89.6 88.7 87.3 86.3 ...
##  $ y : num  65.4 64.9 67.1 68.2 67.9 ...
##  $ z : num  -6417 -6420 -6422 -6424 -6425 ...
```

```
# Converted to list (note class at the bottom)
t.list <- as.list(t)
str( t.list, list.len = 3 )
```

```
## List of 258
##  $ 1   : num [1:39, 1:4] 48 72 96 120 144 168 192 216 240 264 ...
##   ..- attr(*, "dimnames")=List of 2
##   .. ..$ : NULL
##   .. ..$ : chr [1:4] "t" "x" "y" "z"
##  $ 2   : num [1:7, 1:4] 48 72 96 120 144 ...
##   ..- attr(*, "dimnames")=List of 2
##   .. ..$ : NULL
##   .. ..$ : chr [1:4] "t" "x" "y" "z"
##  $ 3   : num [1:19, 1:4] 24 48 72 96 120 144 168 192 216 240 ...
##   ..- attr(*, "dimnames")=List of 2
##   .. ..$ : NULL
##   .. ..$ : chr [1:4] "t" "x" "y" "z"
##   [list output truncated]
##  - attr(*, "class")= chr "list"
```

```
# Convert list back to tracks
str( as.tracks( t.list ), list.len = 3 )
```

```
## List of 258
##  $ 1   : num [1:39, 1:4] 48 72 96 120 144 168 192 216 240 264 ...
##   ..- attr(*, "dimnames")=List of 2
##   .. ..$ : NULL
##   .. ..$ : chr [1:4] "t" "x" "y" "z"
##  $ 2   : num [1:7, 1:4] 48 72 96 120 144 ...
##   ..- attr(*, "dimnames")=List of 2
##   .. ..$ : NULL
##   .. ..$ : chr [1:4] "t" "x" "y" "z"
##  $ 3   : num [1:19, 1:4] 24 48 72 96 120 144 168 192 216 240 ...
##   ..- attr(*, "dimnames")=List of 2
##   .. ..$ : NULL
##   .. ..$ : chr [1:4] "t" "x" "y" "z"
##   [list output truncated]
##  - attr(*, "class")= chr "tracks"
```

```
# Convert dataframe to tracks
str( as.tracks( t.df ), list.len = 3 )
```

```
## List of 258
##  $ 1   : num [1:39, 1:4] 48 72 96 120 144 168 192 216 240 264 ...
##   ..- attr(*, "dimnames")=List of 2
##   .. ..$ : NULL
##   .. ..$ : chr [1:4] "t" "x" "y" "z"
##  $ 10  : num [1:40, 1:4] 24 48 72 96 120 144 168 192 216 240 ...
##   ..- attr(*, "dimnames")=List of 2
##   .. ..$ : NULL
##   .. ..$ : chr [1:4] "t" "x" "y" "z"
##  $ 100 : num [1:11, 1:4] 24 48 72 96 120 144 168 192 216 240 ...
##   ..- attr(*, "dimnames")=List of 2
##   .. ..$ : NULL
##   .. ..$ : chr [1:4] "t" "x" "y" "z"
##   [list output truncated]
##  - attr(*, "class")= chr "tracks"
```

Note that the method `as.tracks.data.frame()` contains arguments `id.column`, `time.column`, and `pos.columns` to specify where information is stored, just like `read.tracks.csv`.

For help, see `?as.list.tracks`, `?as.data.frame.tracks`, `?as.tracks.data.frame`, or `as.tracks.list`.
