# Supplementary material for: CelltrackR: An R package for fast and flexible analysis of immune cell migration data
Source: Immunoinformatics (Amst). Author manuscript; Available in PMC 2023 Apr 6. (PMC10079262; doi:10.1016/j.immuno.2021.100003)
Supplement: Supplemental movies and figures [file NIHMS1884788-supplement-Supplemental_movies_and_figures.zip › SF2-QC.html]

Quality Control and Preprocessing


# Quality Control and Preprocessing

#### Inge Wortel

#### 2021-07-07

# Introduction

Tracking data can be difficult to analyze because of different errors and artifacts that can occur in the data and cause bias in the analysis. This vignette will explain how the package can be used to detect and correct for some of these errors. The topics will be:

1. Checking track lengths and dealing with short tracks
2. Detecting and correcting tissue drift
3. Detecting and correcting tracking errors and imaging artifacts using angle analyses
4. Detecting and correcting variation in timesteps
5. Detecting non-motile cells

This tutorial will outline the tools available along with explanations and examples. For a step-by-step example of the entire workflow, see the vignette on preprocessing the package datasets instead.

# Dataset

First load the package:

```
library( celltrackR )
library( ggplot2 )
```

The package contains two-photon microscopy datasets of B and T cells in a (healthy) cervical lymph node, and of neutrophils responding to an *S. aureus* infection in a mouse ear. These are available in the package under the variable names `TCells`, `BCells`, and `Neutrophils`, e.g.:

```
str( TCells, list.len = 1 )
```

```
## List of 199
##  $ 1     : num [1:39, 1:3] 48 72 96 120 144 168 192 216 240 264 ...
##   ..- attr(*, "dimnames")=List of 2
##   .. ..$ : NULL
##   .. .. [list output truncated]
##   [list output truncated]
##  - attr(*, "class")= chr "tracks"
```

However, in this tutorial we will load the raw, unprocessed T-cell dataset to demonstrate how the package can contribute in preprocessing and quality control.

Load the data (we’ll mostly use the raw T-cell data, but the B cells will come up as well):

```
load( system.file("extdata", "TCellsRaw.rda", package="celltrackR" ) )
load( system.file("extdata", "BCellsRaw.rda", package="celltrackR" ) )
```

The dataset consists of many tracks of individual cells, wrapped up in a tracks object:

```
str( TCellsRaw, list.len = 1 )
```

```
## List of 258
##  $ 1   : num [1:39, 1:4] 48 72 96 120 144 168 192 216 240 264 ...
##   ..- attr(*, "dimnames")=List of 2
##   .. ..$ : NULL
##   .. .. [list output truncated]
##   [list output truncated]
##  - attr(*, "class")= chr "tracks"
```

Each element in this list is a track from a single cell, consisting of a matrix with \((x,y,z)\) coordinates and the corresponding measurement timepoints:

```
head( TCellsRaw[[1]] )
```

```
##        t       x       y        z
## [1,]  48 90.8534 65.3943 -6416.80
## [2,]  72 89.5923 64.9042 -6419.93
## [3,]  96 88.6958 67.1125 -6421.80
## [4,] 120 87.3437 68.2392 -6424.08
## [5,] 144 86.2740 67.9236 -6425.14
## [6,] 168 84.0549 68.2502 -6426.68
```

For the purpose of this tutorial, we’ll just sample a subset from this dataset. Normally you would not do this, but it will help here to speed things up and to avoid cluttered plots; the same methods would apply to a full dataset.

```
# for reproducibility
set.seed(2021)

# sample names, sort them in numeric order, and use them to subset the tracks
Bsample <- sample( names(BCellsRaw),50)
Bsample <- Bsample[ order(as.integer(Bsample)) ]
BCellsRaw <- BCellsRaw[ Bsample ]

# same for the T cells, but now we include a few specific IDs we'll need for the
# analysis below.
Tsample <- sample( names(TCellsRaw),44)
Tsample <- unique( c( Tsample, "5","9658","83","6080","7832","8352" ) )
Tsample <- Tsample[ order(as.integer(Tsample)) ]
TCellsRaw <- TCellsRaw[ Tsample ]
```

# 1 Track length

### 1.1 Finding the length of tracks in the dataset

Especially for in vivo imaging, cells are often imaged in a limited imaging window and for a limited time. This can result in very short tracks that make the data difficult to analyze. We can check the distribution of track lengths in a dataset as follows:

```
# Each track has a coordinate matrix with one row per coordinate;
# The number of steps is the number of rows minus one.
track.lengths <- sapply( TCellsRaw, nrow ) - 1
hist( track.lengths, xlab = "Track length (#steps)", breaks = seq(0,40 ) )
```

```
summary( track.lengths )
```

```
##    Min. 1st Qu.  Median    Mean 3rd Qu.    Max. 
##    6.00    8.00   13.00   17.84   27.00   39.00
```

Alternatively, we can directly check the maximum track length (in number of coordinates, so the number of steps will be this value minus one):

```
# This is the number of coordinates, so the number of steps is one less.
maxTrackLength( TCellsRaw )
```

```
## [1] 40
```

Here, all tracks are at least several steps long. But what if we only want to consider longer tracks?

### 1.2 Dealing with short tracks

To prevent problems from short tracks, we can filter them out using the `filterTracks()` function. The first argument of this function is a function we that returns `TRUE` or `FALSE` for each track:

```
# nrow() of a track is always the number of steps plus one.
# For steps >= min.steps, we can substitute nrow > min.steps:
filter.criterion <- function( x, min.steps ){
  nrow(x) > min.steps
}

TCells.filtered <- filterTracks( filter.criterion, TCellsRaw, min.steps = 11 )
# Or shorthand: filterTracks( function(x) nrow(x) > min.steps, TCellsRaw )
```

Let’s check the effect of the filtering step:

```
# Find lengths of the filtered dataset
track.lengths.filtered <- sapply( TCells.filtered, nrow ) - 1

# Histograms of track lengths before and after
c( "min length before filter" = min( track.lengths ),
   "min length after filter" = min( track.lengths.filtered ) )
```

```
## min length before filter  min length after filter 
##                        6                       11
```

```
# Check how many tracks are left:
length( TCells.filtered )
```

```
## [1] 31
```

The filtering has removed all tracks of 1-10 steps, leaving 31 out of the 50 sampled tracks in the dataset.

> ! **Note** : Filtering out short tracks can introduce bias in a dataset. See Beltman et al (2009) for details and for step-based analyses as alternative methods to deal with short tracks. See the vignette on analysis methods for an explanation of how to use these methods.

In the following, we’ll use the original, unfiltered data in `TCellsRaw`.

# 2 Detecting and correcting drift

> ! **Note** : The methods suggested below all detect a form of global directionality in a dataset. However, any global directionality may either be an artifact from drift, *or* a true phenomenon in the data (for example because cells are following a chemotactic gradient). To distinguish between the two, it is recommended to detect and correct for drift based on tracks from structures that do not move at all, instead of the tracks from the cells of interest. If such data is absent, the original tracks can be used – but this may also remove any true directional bias present in the data.

### 2.1 Adding artificial tissue drift to the Tcell data

To detect global drift of a tissue of interest, let’s add some drift to the `TCellsRaw` data:

```
# Drift is 0.05 micron/sec in each dimension
drift.speed <- c( 0.05, 0.05, 0.05 )
add.drift <- function( x, drift.vector )
{
  # separate timepoints and coordinates
  tvec <- x[,1]
  coords <- x[,-1]
  
  # compute movement due to drift.
  drift.matrix <- matrix( rep( drift.vector, nrow(coords) ),
                          ncol = ncol(coords), byrow= TRUE )
  drift.matrix <- drift.matrix * tvec
  
  # Add drift to coordinates
  x[,-1] <- coords + drift.matrix
  return(x)
}

# Create data with drift
TCells.drift <- as.tracks( lapply( TCellsRaw, add.drift, drift.speed ) )

# Plot tracks and 'rose plot' with overlayed starting point
par(mfrow=c(1,2) )
plot(TCells.drift, main = "drift", cex = 0 )
plot( normalizeTracks(TCells.drift), main = "drift (rose plot)", cex = 0, col = "gray" )
abline( h = 0 )
abline( v = 0 )
```

This drift clearly affects the tracks, with a global bias in the \((1,1)\) direction. In the following, we’ll look at how to quantify this bias.

### 2.2 Detecting global directionality: hotellingsTest

Textor et al (2011) proposed using Hotelling’s T-square test to detect directionality in a dataset. This test computes displacement vectors of all steps in the data, and tests if the mean displacement vector is significantly different from the null vector.

In using this test, it is important to realise that steps from within a track are not independent: cells usually exhibit some kind of persistent motion at least on a short time scale (that is: the same cell does not tend to go in completely opposite directions at to subsequent timepoints). This means that blindly extracting all steps violates the “independent observations” assumption of Hotelling’s test.

For example, applying this to the `TCellsRaw` dataset:

```
hotellingsTest( TCellsRaw, col = "gray" )
```

```
## 
##  Hotelling's one sample T2-test
## 
## data:  
## T2 = 1.3213, df1 = 2, df2 = 872, p-value = 0.5172
## alternative hypothesis: true location is not equal to c(0,0)
```

we find (as expected) that there is no no evidence for global directionality among T cells in a healthy lymph node. However, applying the same to the `BCellsRaw` dataset:

```
hotellingsTest( BCellsRaw, col = "gray" )
```

```
## 
##  Hotelling's one sample T2-test
## 
## data:  
## T2 = 18.978, df1 = 2, df2 = 1276, p-value = 8.175e-05
## alternative hypothesis: true location is not equal to c(0,0)
```

there seems to be a significant directionality. We know, however, that this bias shouldn’t be there–so what is going on?

This is an example of the problem of steps being correlated. If, by chance, a few more cells happen to go in one direction, on the step-level this shows up as a “significant” effect because the individual steps are treated as independent (inflating the power of the test). If we compare the B and T cells in their persistence time in an autocovariance plot (see the vignette on analysis methods for details), we get:

```
# Compute autocovariance, normalize so the first point lies at 1
Tacov <- aggregate( TCellsRaw, overallDot )
Tacov$value <- Tacov$value / Tacov$value[1]

# The same for B cells:
Bacov <- aggregate( BCellsRaw, overallDot )
Bacov$value <- Bacov$value / Bacov$value[1]

# Compare autocovariances:
plot( Tacov )
points( Bacov, col = "red" )
```

We see here that the B cells are more persistent, and the correlation between step directions (on the y-axis) only disappears after 10 steps or more.

When we only consider steps that are some distance apart using the `step.spacing` argument:

```
hotellingsTest( BCellsRaw, col = "gray", step.spacing = 10 )
```

```
## 
##  Hotelling's one sample T2-test
## 
## data:  
## T2 = 0.962, df1 = 2, df2 = 135, p-value = 0.6214
## alternative hypothesis: true location is not equal to c(0,0)
```

there is no longer evidence for directionality. (Note, however, that the power of the test is also reduced because the larger step spacing reduces the total number of steps).

By contrast, directionality remains obvious in the dataset with drift, even at a large step spacing:

```
hotellingsTest( TCells.drift, plot = TRUE, col = "gray", step.spacing = 10 )
```

```
## 
##  Hotelling's one sample T2-test
## 
## data:  
## T2 = 36.856, df1 = 2, df2 = 99, p-value = 1.799e-07
## alternative hypothesis: true location is not equal to c(0,0)
```

Here we also visualize the effect by setting `plot=TRUE`; the blue ellipse indicates the global direction (which is indeed the direction we simulated drift in).

> ! **Note** : The appropriate value of `step.spacing` depends on the level of persistence expected in the cells of interest, as well as on the time resolution of the experiment. An autocorrelation or autocovariance plot may provide insights in the persistence time for the cells of interest (see the vignette on analysis methods for details). If tracks of static structures are used to detect and correct for drift – as is recommended to distinguish between drift and truly directed movement – a `step.spacing` of zero is safe to use because there should be no inherent persistence for something that does not move.

Of note, by default `hotellingsTest()` is performed on a *projection* of the tracks on only the \(x\) and \(y\) dimensions. For 3D tracking data, we can specify that all three dimensions should be taken into account (although plotting is not supported in 3D):

```
hotellingsTest( TCellsRaw, dim = c("x","y","z"), step.spacing = 10 )
```

```
## 
##  Hotelling's one sample T2-test
## 
## data:  
## T2 = 1.5623, df1 = 3, df2 = 98, p-value = 0.6761
## alternative hypothesis: true location is not equal to c(0,0,0)
```

```
hotellingsTest( TCells.drift, dim = c("x","y","z"), step.spacing = 10 )
```

```
## 
##  Hotelling's one sample T2-test
## 
## data:  
## T2 = 47.594, df1 = 3, df2 = 98, p-value = 2.396e-08
## alternative hypothesis: true location is not equal to c(0,0,0)
```

However, this is not always recommended; the z-dimension in many two-photon experiments is much smaller than the x and y dimensions, and the resolution in this dimension is smaller as well. The z-coordinates of these data are therefore not always reliable and it can be better to leave them out for some track analyses.

### 2.3 Detecting global directionality: angle analysis

Beltman et al (2009) proposed an analysis of angles versus distance between cell pairs to detect global directionality in a dataset. Use the function `analyzeCellPairs` to get a dataframe with for each pair of tracks in the dataset the angle (between their displacement vectors) and the distance (min distance between the tracks at any timepoint). Note that the distance is not defined for pairs of tracks that have no overlap in timepoints – these will get an NA value.

```
# compute for both original as drift data
df.drift <- analyzeCellPairs( TCells.drift )
df.norm <- analyzeCellPairs( TCellsRaw )

# Plot
p.norm <- ggplot( df.norm, aes( x = dist, y = angle ) ) +
  geom_point( color = "gray70", size = 0.5 ) +
  stat_smooth( span = 1, fill = "blue" )+
  labs( x = "distance between cell pairs",
        y = "angle between cell pairs",
        title = "original data") +
  geom_hline( yintercept = 90, color = "red" ) +
  theme_classic()

p.drift <- ggplot( df.drift, aes( x = dist, y = angle ) ) +
  geom_point( color = "gray70", size = 0.5 ) +
  stat_smooth( span = 1, fill = "blue" )+
  labs( x = "distance between cell pairs",
        y = "angle between cell pairs",
        title = "data with drift") +
  geom_hline( yintercept = 90, color = "red" ) +
  theme_classic()

gridExtra::grid.arrange( p.norm, p.drift, ncol = 2 )
```

In the original data, the mean angle is roughly 90 degrees on average for cell pairs at any distance from each other, which is what we would expect if there is no global directionality in the data. In the data with drift, the average angle is lower than we would expect even at large distances between cell pairs – which indicates that there is global directionality (even cells far apart move roughly in the same direction).

We can perform a similar analysis on the level of single steps to boost the power. The function `analyzeStepPairs()` finds all pairs of steps from *different* cells that occur at the *same* timepoint, and then computes distances (between the step starting points) and angles between them. Note that we use a function in the argument `filter.steps` to take into account only steps with a minimum displacement, to avoid noise from steps where the cell is pausing.

```
# compute for both original as drift data.
df.drift <- analyzeStepPairs( TCells.drift, filter.steps = function(x) displacement(x)>2  )
df.norm <- analyzeStepPairs( TCellsRaw, filter.steps = function(x) displacement(x)>2  )

# Plot
p.norm <- ggplot( df.norm, aes( x = dist, y = angle ) ) +
  geom_point( color = "gray70", size = 0.5 ) +
  stat_smooth( span = 0.75, fill="blue" )+
  labs( x = "distance between step pairs",
        y = "angle between step pairs",
        title = "original data") +
  geom_hline( yintercept = 90, color = "red" ) +
  theme_classic()

p.drift <- ggplot( df.drift, aes( x = dist, y = angle ) ) +
  geom_point( color = "gray70", size = 0.5 ) +
  stat_smooth( span = 0.75,  fill="blue")+
  labs( x = "distance between step pairs",
        y = "angle between step pairs",
        title = "data with drift") +
  geom_hline( yintercept = 90, color = "red" ) +
  theme_classic()

gridExtra::grid.arrange( p.norm, p.drift, ncol = 2 )
```

Again we see a mean step pair angle that is below 90 degrees for the data with drift, which is not the case for the original data.

### 2.4 Correcting drift

If Hotelling’s test and/or angle analyses provide evidence for drift in the dataset, this can be corrected by computing the mean step displacement:

```
# Get steps and find their displacement vectors
steps.drift <- subtracks( TCells.drift, 1 )
step.disp <- t( sapply( steps.drift, displacementVector ) )

# Get the mean
mean.displacement <- colMeans( step.disp )

# Divide this by the mean timestep to get a drift speed
drift.speed <- mean.displacement/timeStep( TCells.drift )
drift.speed
```

```
## [1] 0.05411294 0.04793798 0.04538616
```

This is indeed roughly the drift speed we introduced in the data. Now remove this:

```
correct.drift <- function( x, drift.vector )
{
  # separate timepoints and coordinates
  tvec <- x[,1]
  coords <- x[,-1]
  
  # compute movement due to drift.
  drift.matrix <- matrix( rep( drift.vector, nrow(coords) ),
                          ncol = ncol(coords), byrow= TRUE )
  drift.matrix <- drift.matrix * tvec
  
  # Add drift to coordinates
  x[,-1] <- coords - drift.matrix
  return(x)
}

# Create data with drift
TCells.corrected <- as.tracks( lapply( TCells.drift, correct.drift, drift.speed ) )

# Compare (zoom in on part of the field to see better)
par( mfrow = c(1,2) )
plot( TCellsRaw, col = "blue", main = "uncorrected", cex = 0, pch.start = NA, xlim = c(200,400), ylim = c(0,200) )
plot( TCells.drift, col = "red", add = TRUE, cex = 0, pch.start = NA )
plot( TCellsRaw, col = "blue", main = "corrected", cex = 0, pch.start = NA, xlim = c(200,400), ylim = c(0,200)  )
plot( TCells.corrected, col = "red", add = TRUE, cex = 0, pch.start = NA  )
```

While the correction is not perfect, at least the tracks resemble their original shape more.

# 3 Detecting artifacts using angle analyses

### 3.1 Detecting double tracking: angle versus distance between cell pairs

To simulate a dataset with double tracking, take one track and keep it roughly the same, but with some small noise added to the coordinates:

```
# Take the track with id "5"
dup.track <- TCellsRaw[["5"]]

# Add some noise to coordinates
dup.track[,"x"] <- dup.track[,"x"] + rnorm( nrow(dup.track), sd = 0.5 )
dup.track[,"y"] <- dup.track[,"y"] + rnorm( nrow(dup.track), sd = 0.5 )
dup.track[,"z"] <- dup.track[,"z"] + rnorm( nrow(dup.track), sd = 0.5 )

# Wrap the track in a tracks object and add it to the TCell data with
# a unique id number
dup.track <- wrapTrack( dup.track )
new.id <- max( as.numeric( names( TCellsRaw ) ) ) + 1
names(dup.track) <- as.character( new.id )
TCells.dup <- c( TCellsRaw, dup.track )
```

This can again be detected by plotting the angle between cell pairs versus the distance between cell pairs (see also section 2.3 above and Beltman et al (2009)):

```
df <- analyzeCellPairs( TCells.dup )

# label cellpairs that have both angle and distance below threshold
angle.thresh <- 5 # in degrees
dist.thresh <- 10 # this should be the expected cell radius
df$id <- paste0( df$cell1,"-",df$cell2 )
df$id[ !(df$angle < angle.thresh & df$dist < dist.thresh) ] <- "" 

# Plot; zoom in on the region with small angles and distances
ggplot( df, aes( x = dist, y = angle ) ) +
  geom_point( color = "gray40" ) +
  geom_text( aes( label = id ), color = "red", hjust = -0.1 ) +
  labs( x = "distance between cell pairs",
        y = "angle between cell pairs" ) +
  coord_cartesian( xlim=c(0,30), ylim=c(0,20) ) +
  geom_hline( yintercept = angle.thresh, col = "blue",lty=2 ) +
  geom_vline( xintercept = dist.thresh, col = "blue", lty=2) +
  theme_classic()
```

We indeed find the pair of tracks with ids “5” (the original track) and “9659” (the noisy duplicate of the original track). Plot the suspected tracks specifically to check:

```
par( mfrow=c(1,3))
plot( TCells.dup[c("5","9659") ] )
plot( TCells.dup[c("83","6080") ] )
plot( TCells.dup[c("7832","8352") ] )
```

Only the first really looks like double tracking; so we can remove the track with ID “9659”:

```
corrected.dup <- TCells.dup[ names( TCells.dup ) != "9659" ]
```

### 3.2 Detecting tracking errors near border or imprecise z-calibration: distances and angles to border planes

A plot of angles and distances to the border planes of the imaging volume can help detect artifacts (Beltman et al (2009) ). We can compute these for each step using the functions `distanceToPlane` and `angleToPlane`.

To specify a border plane, we first need three points on that plane. We can either define the imaging volume manually or estimate it using `boundingBox`:

```
tracks <- TCellsRaw
bb <- boundingBox( tracks )
bb
```

```
##       t     x       y        z
## min  24   0.4   0.400 -6441.29
## max 960 409.2 383.001 -6381.29
```

Let’s take the two borders in the z-dimension as an example. The lower z-plane contains points (minx,miny,minz), (maxx,miny,minz), (maxx,maxy,minz). The upper z-plane is at distance (maxz-minz) from the lower plane.

```
# Define points:
lower1 <- c( bb["min","x"], bb["min","y"], bb["min","z"] )
lower2 <- c( bb["max","x"], bb["min","y"], bb["min","z"] )
lower3 <- c( bb["max","x"], bb["max","y"], bb["min","z"] )
zsize <- bb["max","z"] - bb["min","z"]

# Compute angles and distances of steps to this plane.
single.steps <- subtracks( tracks, 1 )
angles <- sapply( single.steps, angleToPlane, p1 = lower1, 
                  p2 = lower2, p3 = lower3 )
distances <- sapply( single.steps, distanceToPlane, p1 = lower1,
                     p2 = lower2, p3 = lower3 )
df <- data.frame( angles = angles,
                  distances = distances )

# Plot
ggplot( df, aes( x = distances,
                 y = angles ) ) +
  geom_point( color = "gray70", size = 0.5 ) +
  stat_smooth( method = "loess", span = 1, fill="blue" ) +
  geom_hline( yintercept = 32.7, color = "red" ) +
  scale_x_continuous( limits=c(0,zsize ), expand = c(0,0) ) +
  theme_classic()
```

```
## `geom_smooth()` using formula 'y ~ x'
```

The mean angle to the border plane should be roughly 32.7 degrees (the red horizontal line). Tracking errors near the border plane would result in a lower average angle near the left and right side of the plots, which is indeed what we see here. This plot also seems to indicate imprecise z-calibration, which results in a systematic deviation from the 32.7 degree angle at any distance to the plane (e.g. at the middle distances in this plot) (Beltman et al (2009) ).

When we plot the data in the XZ instead of the XY plane, we can see the problem:

```
par( mar = c(5, 4, 0.5, 2) + 0.1 )
plot( TCellsRaw, dims = c("x","z" ) )
```

We see here that many tracks seem to go parallel to the z-border for quite some time. This is indeed a tracking error, and it is worsened in this dataset because the z-dimension is much smaller than the x and y dimensions:

```
boundingBox( TCellsRaw )
```

```
##       t     x       y        z
## min  24   0.4   0.400 -6441.29
## max 960 409.2 383.001 -6381.29
```

In such cases, it is often better to work with tracks projected on the XY-plane; see the last section of this tutorial, where we describe preprocessing choices made for the datasets included with the package.

# 4 Detecting and correcting variation in time resolution

### 4.1 Detecting variation in timesteps

Track analysis methods usually assume a constant time interval \(\Delta t\) between consecutive images in a time-lapse microscopy dataset. In reality, there are mostly at least small fluctuations in the \(\Delta t\) between consecutive images. To find the \(\Delta t\) of each step, we first extract single steps using the `subtracks()` function (see also the vignette on analysis methods):

```
# Extract all subtracks of length 1 (that is, all "steps")
single.steps <- subtracks( TCellsRaw, 1 )

# The output is a new tracks object with a unique track for each step
# in the data (no longer grouped by the original cell they came from):
str( single.steps, list.len = 3 )
```

```
## List of 874
##  $ 5.1    : num [1:2, 1:4] 24 48 168.81 166.21 5.18 ...
##   ..- attr(*, "dimnames")=List of 2
##   .. ..$ : NULL
##   .. ..$ : chr [1:4] "t" "x" "y" "z"
##  $ 5.2    : num [1:2, 1:4] 48 72 166.21 161.71 4.14 ...
##   ..- attr(*, "dimnames")=List of 2
##   .. ..$ : NULL
##   .. ..$ : chr [1:4] "t" "x" "y" "z"
##  $ 5.3    : num [1:2, 1:4] 72 96 161.7 161.2 9.8 ...
##   ..- attr(*, "dimnames")=List of 2
##   .. ..$ : NULL
##   .. ..$ : chr [1:4] "t" "x" "y" "z"
##   [list output truncated]
##  - attr(*, "class")= chr "tracks"
```

After extracting the single steps, we can find the median \(\Delta t\) using the `timeStep()` function:

```
median.dt <- timeStep( TCellsRaw )
median.dt
```

```
## [1] 24
```

We then find the actual \(\Delta t\) of each step by applying the `duration()` function to each step in the `single.steps` object:

```
step.dt <- sapply( single.steps, duration )
str(step.dt)
```

```
##  Named num [1:874] 24 24 24 24 24 24 24 24 24 24 ...
##  - attr(*, "names")= chr [1:874] "5.1" "5.2" "5.3" "5.4" ...
```

And visualize the difference with the median \(\Delta t\) in a histogram (expressed as a percentage of \(\Delta t\):

```
dt.diff.perc <- (step.dt - median.dt) * 100 / median.dt
hist( dt.diff.perc, xlab = "dt (percentage difference from median)" )
```

We see that most of the fluctuations are small, on the left end of the histogram, but from the range of the histogram there also seem to be larger fluctations. Indeed, if we look at the range and unique values of step durations:

```
range( step.dt )
```

```
## [1] 24 48
```

```
unique( step.dt )
```

```
## [1] 24 48
```

then we see that as expected, the minimum step duration is 24 seconds – but there are apparently also steps of 48 seconds. Let’s see how many:

```
sum( step.dt == 48 )
```

```
## [1] 1
```

Thus, there are apparently six places somewhere in the tracks where a coordinate is missing. These “gaps” need fixing: if steps take twice as long, then step-based metrics such as displacements and turning angles will be affected in downstream analyses. In the following, we’ll discuss this problem in more detail.

### 4.2 Example: detecting missing data in tracks

To demonstrate the effect of gaps in tracks, we will exacerbate the problem by removing some timepoints in a few tracks in the `TCellsRaw` data:

```
# This function randomly removes coordinates from a track dataset with probability "prob"
remove.points <- function( track, prob=0.1 ){
  
    tlength <- nrow( track )
    remove.rows <- sample( c(TRUE,FALSE), tlength, replace=TRUE,
                           prob = c(prob, (1-prob) ) )
    track <- track[!remove.rows,]
  return(track)
}

# Apply function to dataset to randomly remove coordinates in the data
TCells.gap <- as.tracks( lapply( TCellsRaw, remove.points ) )
```

This (now exacerbated) artifact has an effect on for example the step-based displacement:

```
# duration of the individual steps
steps.gap <- subtracks( TCells.gap, 1 )

T1.step.disp <- sapply( single.steps, displacement )
T1.gap.disp <- sapply( steps.gap, displacement )

lapply( list( original = T1.step.disp, gaps = T1.gap.disp ), summary )
```

```
## $original
##    Min. 1st Qu.  Median    Mean 3rd Qu.    Max. 
##  0.0508  1.2591  2.3938  3.3663  4.0802 22.2281 
## 
## $gaps
##     Min.  1st Qu.   Median     Mean  3rd Qu.     Max. 
##  0.06119  1.30953  2.39739  3.50125  4.28424 22.22807
```

Note that the mean and median displacement are slightly higher due to the gaps, because steps with a gap in between are actually two steps and thus have a larger displacement.

For a simple step-based analysis of displacement, we can use `normalizeToDuration()` to correct for differences in time resolution:

```
T1.norm.disp <- sapply( single.steps, normalizeToDuration( displacement ) )
T1.norm.gap.disp <- sapply( steps.gap, normalizeToDuration( displacement ) )
lapply( list( original = T1.norm.disp, gaps = T1.norm.gap.disp ), summary )
```

```
## $original
##     Min.  1st Qu.   Median     Mean  3rd Qu.     Max. 
## 0.002117 0.052462 0.099740 0.139947 0.170008 0.926170 
## 
## $gaps
##    Min. 1st Qu.  Median    Mean 3rd Qu.    Max. 
## 0.00255 0.05092 0.09727 0.13494 0.16612 0.92617
```

The difference in displacements is now gone. For more complicated analyses, one might want to correct the gaps in the dataset via splitting tracks or via interpolation – see the next section.

### 4.3 Correcting gaps or variation in timesteps

If a dataset contains gaps, as detected in the histogram of \(\Delta t\), we can correct these using the `repairGaps` function, where we can select one of three correction methods using the `how` argument:

1. Dropping all tracks with gaps (`how = "drop"`)
2. Splitting tracks around the gaps(`how = "split"`)
3. Interpolating the track (`how = "interpolate"`)

For example:

```
# Repair gaps by splitting or interpolation, the number of tracks is 
# different after each fix
split.gap <- repairGaps( TCells.gap, how = "split" )
interpolate.gap <- repairGaps( TCells.gap, how = "interpolate" )

c( "after splitting" = length( split.gap),
   "after interpolation" = length( interpolate.gap ) )
```

```
##     after splitting after interpolation 
##                 108                  49
```

### 4.4 Comparing experiments with a different time resolution

Finally, if we wish to compare tracks from experiments imaged at a different time resolution \(\Delta t\), we can use the function `interpolateTrack()` to estimate the position of each cell at any set of timepoints of interest.

For example, let’s create a second T cell dataset where we keep only every second timepoint (to effectively increase \(\Delta t\) twofold while keeping the same dataset):

```
T2 <- subsample( TCellsRaw, k = 2 )
```

When we now perform a step-based analysis of displacement, the two will be different:

```
# displacement
T1.steps <- subtracks( TCellsRaw, 1 )
T1.disp <- sapply( T1.steps, displacement )

T2.steps <- subtracks( T2, 1 )
T2.disp <- sapply( T2.steps, displacement )

lapply( list( T1 = T1.disp, T2 = T2.disp ), summary )
```

```
## $T1
##    Min. 1st Qu.  Median    Mean 3rd Qu.    Max. 
##  0.0508  1.2591  2.3938  3.3663  4.0802 22.2281 
## 
## $T2
##    Min. 1st Qu.  Median    Mean 3rd Qu.    Max. 
##   0.001   1.668   3.651   5.149   7.157  26.682
```

To correct for the difference in time resolution, we can interpolate both datasets at a fixed time resolution:

```
# interpolate both datasets at the time resolution of the neutrophils
dt <- timeStep( TCellsRaw )
interpolate.dt <- function( x, dt, how = "spline" ){
  trange <- range( timePoints( wrapTrack( x ) ) )
  tvec <- seq( trange[1], trange[2], by = dt )
  x <- interpolateTrack( x, tvec, how = how )
  return(x)
}

T1.corrected <- as.tracks( lapply( TCellsRaw, interpolate.dt, dt = dt ) )
T2.corrected <- as.tracks( lapply( T2, interpolate.dt, dt = dt ) )

# Check the effect on the displacement statistics:
T1.corr.steps <- subtracks( T1.corrected, 1 )
T1.corr.disp <- sapply( T1.corr.steps, displacement )

T2.corr.steps <- subtracks( T2.corrected, 1 )
T2.corr.disp <- sapply( T2.corr.steps, displacement )

lapply( list( T1 = T1.disp, T2 = T2.disp, 
              T1.corr = T1.corr.disp, T2.corr = T2.corr.disp ), 
        summary )
```

```
## $T1
##    Min. 1st Qu.  Median    Mean 3rd Qu.    Max. 
##  0.0508  1.2591  2.3938  3.3663  4.0802 22.2281 
## 
## $T2
##    Min. 1st Qu.  Median    Mean 3rd Qu.    Max. 
##   0.001   1.668   3.651   5.149   7.157  26.682 
## 
## $T1.corr
##    Min. 1st Qu.  Median    Mean 3rd Qu.    Max. 
##  0.0508  1.2591  2.3940  3.3628  4.0873 22.2281 
## 
## $T2.corr
##    Min. 1st Qu.  Median    Mean 3rd Qu.    Max. 
##  0.0908  0.9137  1.9706  2.7592  3.7231 14.8516
```

The difference is now much smaller.

### 5 Detecting non-motile cells

Finally, let’s look again at the T-cell tracks in the raw data; for the following, we’ll use the full dataset again rather than a sample:

```
load( system.file("extdata", "TCellsRaw.rda", package="celltrackR" ) )

par( mar = c(2, 2, 1, 1) + 0.1 )
plot( TCellsRaw )
```

We see here that there are quite some cells that do not seem to move at all (e.g. on the top of the plot, but also in other places).

Non-motile cells can be there for different reasons:

- Because they are dead (e.g. from photoxicity in the experiment);
- As part of their migration mode (e.g. prolonged pauses in between movements);
- For other (biological) reasons; for example, our T cells in the lymph node may stop when they engage with antigen.

Whether or not such cells should be kept in the data analysis depends on context. For example, dead cells should probably be filtered out, but this does not apply if pausing is part of the motility mechanism. It also depends on the exact question, but for now, let’s assume we wish to filter out non-motile cells before downstream motility analysis.

### 5.1 Option 1: filtering based on track measures such as speed

One option is to filter cells based on certain statistics, such as speed. For example, we can make a speed histogram:

```
cellSpeeds <- sapply( TCellsRaw, speed )
hist( cellSpeeds, breaks = 30 )
```

We can then set some cutoff, and filter based on that:

```
cutoff <- 0.1 # 0.1 micron/sec = 6 micron/min
loSpeed <- filterTracks( function(t) speed(t) < cutoff, TCellsRaw )
hiSpeed <- filterTracks( function(t) speed(t) >= cutoff, TCellsRaw )
```

Let’s visualize both:

```
par(mfrow=c(1,2))
plot( loSpeed, main = "Below cutoff" )
plot( hiSpeed, main = "Above cutoff" )
```

We see, however, that this does not completely work: e.g. the cell in the “above cutoff” (at \(x \approx 300, y \approx 380\)) apparently has a high speed, but does not seem to be moving if we look at the track. Paradoxically, if a cell just “jiggles” in place, it can still have a relatively high speed. Likewise, some of the cells below the cutoff do seem to show some motion (e.g. the cells in the middle of the field); this can happen when cell speed is heterogeneous (e.g. because the cell alternates between motile and less motile phases).

A threshold like this can still be an option, but it also leaves us with the choice of how to choose this threshold – especially since we don’t clearly see two populations in the speed histogram. In the following, we will therefore describe another option.

### 5.2 Option 2: modelling coordinates as multivariate Gaussian

Our second option is to consider the \((x,y)\) (or \(x,y,z\)) coordinates of the cells. A cell that is not really motile will only “jiggle in place” due to measurement error or passive Brownian motion. By contrast, for a “motile” cell, we expect it to displace at least some distance from its starting point at some point during the track.

This leaves us with two “models”: - For a non-motile cell, we expect that the \(x,y\) coordinates are relatively well-described by bivariate Gaussian noise around the mean position; - For a motile cell, we expect that this is not the case, and that a model of 2 (or more) Gaussians along the track will describe the data better than a single Gaussian model.

For each cell, we can then compare these models using the (Bayesian Information Criterion (BIC))[https://en.wikipedia.org/wiki/Bayesian\_information\_criterion]:

\[\text{BIC} = k \log n - 2 \log \hat{L}\] where \(k\) is the number of model parameters, \(n\) is the number of observations, and \(\hat{L}\) the likelihood of the model.

We can then make a decision based on the \(\Delta\text{BIC}\) between our two models:

\[\Delta\text{BIC} = \text{BIC}\_\text{nonMotile} - \text{BIC}\_\text{motile}\] According to general guidelines of the BIC, we can then say that there is evidence that cells are better described by the “motile” model if \(\Delta\text{BIC} \geq 6\).

First, let’s implement the BIC for the non-motile model:

```
bicNonMotile <- function( track, sigma ){
  
  # we'll use only x and y coordinates since we saw earlier that the z-dimension was
  # not so reliable
  allPoints <- track[,c("x","y")]
  
  # Compute the log likelihood under a multivariate gaussian.
  # For each point, we get the density under the Gaussian distribution 
  # (using dmvnorm from the mvtnorm package).
  # The product of these densities is then the likelihood; but since we need the 
  # log likelihood, we can also first log-transform and then sum:
  Lpoints <- mvtnorm::dmvnorm( allPoints, 
                      mean = colMeans(allPoints), # for a Gaussian around the mean position
                      sigma = sigma*diag(2), # sd of the Gaussian (which we should choose)
                      log = TRUE )
  logL <- sum( Lpoints )
  
  # BIC = k log n - 2 logL; here k = 3 ( mean x, mean y, sigma )
  return( 3*log(nrow(allPoints)) - 2*logL )
}
```

Likewise, we need a BIC for the motile model. There we need to also choose the cutoff point \(m\), the point at which to split track coordinates between the two Gaussians we will fit:

```
# the BIC for a given cutoff m
bicAtCutoff <- function( track, m, sigma ){
  
  # we'll use only x and y coordinates since we saw earlier that the z-dimension was
  # not so reliable
  allPoints <- track[,c("x","y")]
  
  # Split into two coordinate sets based on the cutoff m:
  firstCoords <- allPoints[1:m, , drop = FALSE]
  lastCoords <- allPoints[(m+1):nrow(allPoints), , drop = FALSE ]
  
  # Compute log likelihood under two separate Gaussians:
  Lpoints1 <- mvtnorm::dmvnorm( firstCoords, 
    mean = colMeans(firstCoords), 
    sigma = sigma*diag(2), 
    log = TRUE )
  Lpoints2 <- mvtnorm::dmvnorm( lastCoords, 
    mean = colMeans(lastCoords), 
    sigma = sigma*diag(2), 
    log = TRUE )
  logL <- sum( Lpoints1 ) + sum( Lpoints2 )
  
  # BIC = k log n - 2 logL; here k = 6 ( 2*mean x, 2*mean y, sigma, and m )
  return( 6*log(nrow(allPoints)) - 2*logL )
}

# We'll try all possible cutoffs m, and choose best model (minimal BIC)
# to compare to our non-motile "null hypothesis":
bicMotile <- function( track, sigma ){
  
  # cutoff anywhere from after the first two coordinates to 
  # before the last two (we want at least 2 points in each Gaussian,
  # to prevent fitting of a single point)
  cutoffOptions <- 2:(nrow(track)-2)
  
    min( sapply( cutoffOptions, function(m) bicAtCutoff(track,m,sigma) ) )
}
```

Finally, let’s use this to compute the \(\Delta\text{BIC}\) for the different cells. We will choose \(\sigma = 7\mu\)m, which means that we will consider as ‘non-motile’ any cell that can be described by a Gaussian of roughly the cell size. Only cells that deviate notably from this (\(\Delta\text{BIC} \geq 6\)) will be considered motile:

```
deltaBIC <- function( x, sigma ){
  b1 <- bicNonMotile( x, sigma )
  b2 <- bicMotile( x, sigma )
  d <- b1 - b2
  d
}

dBIC <- sapply( TCellsRaw, deltaBIC, 7 )
motile <- TCellsRaw[ dBIC >= 6 ]
nonMotile <- TCellsRaw[ dBIC < 6 ]


# Plot again for comparison:
par(mfrow=c(1,2))
plot( motile, main = "Motile" )
plot( nonMotile, main = "Non-Motile" )
```

Although it remains impossible to separate motile and non-motile cells perfectly, at least the non-motile cells really seem not to be moving, and most of the motile cells *do* seem to be moving. Interesting, if we compare this \(\Delta\text{BIC}\) to the speed of the same cell:

```
plot( dBIC, cellSpeeds, xlim = c(0,20) )
abline( v = 6, col = "red" ) # delta BIC cutoff
abline( h = max(cellSpeeds), col = "blue", lty = 2 ) # max speed of all cells
```

we see again that some of the cells that are clearly non-motile based on their track can still have quite high speeds, suggesting that a cutoff based on speed indeed would not have been a good filter here.
