# Supplementary material for: CelltrackR: An R package for fast and flexible analysis of immune cell migration data
Source: Immunoinformatics (Amst). Author manuscript; Available in PMC 2023 Apr 6. (PMC10079262; doi:10.1016/j.immuno.2021.100003)
Supplement: Supplemental movies and figures [file NIHMS1884788-supplement-Supplemental_movies_and_figures.zip › SF3-data-QC.html]

Quality Control and Preprocessing of the Datasets in the Package


# Quality Control and Preprocessing of the Datasets in the Package

#### Inge Wortel

#### 2021-07-07

## Introduction

This tutorial will apply the tools from the vignette on quality control to clean up the raw data of three two-photon microscopy datasets shipped with the package: tracks of B and T cells in a (healthy) murine cervical lymph node, and of neutrophils responding to an *S. aureus* infection in a mouse ear. The preprocessed data are available in the package under the variable names `TCells`, `BCells`, and `Neutrophils`, but here we will show how these were obtained from the raw data.

## Before we start

First load the package:

```
library( celltrackR )
library( ggplot2 )
```

And load the raw data of each set so we can do the preprocessing from scratch:

```
load( system.file("extdata", "TCellsRaw.rda", package="celltrackR" ) )
load( system.file("extdata", "BCellsRaw.rda", package="celltrackR" ) )
load( system.file("extdata", "NeutrophilsRaw.rda", package="celltrackR" ) )
```

In the following, we will perform QC and preprocessing on these data to generate the `TCells`, `BCells`, and `Neutrophils` data in the package.

## 1 Short tracks

Let’s look at the minimal track lengths in the three datasets:

```
# nrow on a track gives # coordinates; number of steps is this minus one
minStepsT <- min( sapply( TCellsRaw, nrow ) - 1 )
minStepsB <- min( sapply( BCellsRaw, nrow ) - 1 )
minStepsN <- min( sapply( NeutrophilsRaw, nrow ) - 1 )
c( "T cells" = minStepsT, "B cells" = minStepsB, "Neutrophils" = minStepsN )
```

```
##     T cells     B cells Neutrophils 
##           6           6           1
```

We see that the B and T cells have tracks from at least 6 steps, but the neutrophils have a minimum track length of a single step. This will give us problems with downstream analysis. Although we don’t want to filter too stringently on track length (that would introduce biases), let’s see what percentage of tracks has fewer than 3 steps (4 coordinates):

```
veryShort <- sum( sapply( NeutrophilsRaw, nrow ) < 4 )
100 * veryShort / length( NeutrophilsRaw )
```

```
## [1] 9.325397
```

This is less than 10%; so for our example dataset let’s just remove them (for a real analysis, it might be worthwhile to check these short tracks further).

```
Neutrophils <- filterTracks( function(t) nrow(t) >= 4, NeutrophilsRaw )
TCells <- TCellsRaw
BCells <- BCellsRaw
```

## 2 Drift?

To check for possible drift, we apply Hotelling’s test with appropriate step spacing (see section 2.2 in the vignette on quality control methods for details):

```
hotellingsTest( TCells, step.spacing = 10 )
```

```
## 
##  Hotelling's one sample T2-test
## 
## data:  
## T2 = 1.2923, df1 = 2, df2 = 550, p-value = 0.5251
## alternative hypothesis: true location is not equal to c(0,0)
```

```
hotellingsTest( BCells, step.spacing = 10 )
```

```
## 
##  Hotelling's one sample T2-test
## 
## data:  
## T2 = 0.75818, df1 = 2, df2 = 265, p-value = 0.6858
## alternative hypothesis: true location is not equal to c(0,0)
```

```
hotellingsTest( Neutrophils, step.spacing = 10 )
```

```
## 
##  Hotelling's one sample T2-test
## 
## data:  
## T2 = 114.09, df1 = 2, df2 = 725, p-value < 2.2e-16
## alternative hypothesis: true location is not equal to c(0,0)
```

As expected, we find no evidence of global directionality in the T-cell and B-cell datasets (which should be following a more or less random walk in the uninfected lymph node). We do find this in the Neutrophil dataset – but that is a true effect rather than an artifact since Neutrophils are actually being attracted by an infection. We therefore do not perform any drift correction on these datasets.

## 3 Border artifacts?

All datasets clearly have border artifacts in the z-dimension, given the limited depth of this dimension (see section 3 in the vignette on quality control methods for details):

```
par( mfrow=c(3,1), mar = c(0,0,0,0) + 0.1 )
plot( TCells, dims = c("x","z"), xaxt='n', yaxt = 'n', ann=FALSE )
plot( BCells, dims = c("x","z"), xaxt='n', yaxt = 'n', ann=FALSE )
plot( Neutrophils, dims = c("x","z"), xaxt='n', yaxt = 'n', ann=FALSE )
```

Note the very straight horizontal tracks at the top and bottom. As this artifact affects many of the cells, we cannot simple remove them; this would introduce a bias since we are more likely to remove faster cells that reach the border during the experiment. Instead, we’ll deal with this by projecting all tracks on the XY plane, discarding the z-coordinate.

Still, we have similar artifacts at the x and y borders. E.g. for the T cells:

```
# zoom in on border cells
plot( TCells, xlim = c(400, 420), ylim = c(250,350))
```

These are much fewer cells, so let’s just remove those based on their angles and distances to the x and y borders. To assist us, we can define the following functions:

```
# Checks angle of a cell's steps to the borders
# (bb is the bounding box of all tracks, used to define those borders)
# returns the fraction of a cell's steps that are aligned with 
# one of the borders
angleCheck <- function( steps, bb, thresholdAngle = 0.1 ){
    # only consider x and y borders since filtering on the z-border would
  # remove too many cells (we'll later project on the xy plane instead):
    minx <- bb["min","x"]
    miny <- bb["min","y"]
    
    angles <- matrix( 0, nrow = length(steps), ncol = 2 )
    angles[,1] <- sapply( steps, angleToPlane, 
                          p1 = c(minx,0,1), p2=c(minx,1,0), p3 = c(minx,0,0) )
    angles[,2] <- sapply( steps, angleToPlane, 
                          p1 = c(0,miny,1), p2=c(1,miny,0), p3 = c(0,miny,0) )

    minAng <- apply( angles, 1, min, na.rm = TRUE )
    maxAng <- apply( angles, 1, max, na.rm = TRUE )

    # Steps are suspect if they are at angle ~0 or ~180 to the border plane.
    return( sum( minAng < thresholdAngle | maxAng > (180-thresholdAngle) )/length(steps) )
}

# Checks distance of a cell's steps to the borders; returns the fraction of steps
# that are closer than a certain threshold to one of the borders.
distanceCheck <- function( steps, bb, threshold = 1 ){
    total <- numeric( length(steps) )
    for( d in c("x","y") ){
        # distance to the lower border
        minDist <- sapply( steps, function(x) min( abs( x[,d] - bb["min",d] ) ) )
        
        # distance to the higher border
        maxDist <- sapply( steps, function(x) min( abs( x[,d] - bb["max",d] ) ) )
        
        # suspect if one of these distances is below threshold
        nearBorder <- ( minDist < threshold ) | ( maxDist < threshold )     
        total[nearBorder] <- 1
    }
    return( sum(total)/length(total) )  
}

# Remove tracks that have more than maxFrac steps that are aligned with the border AND 
# within a certain distance to the border:
notAtBorder <- function( tracks, angleThreshold = 0.1, distanceThreshold = 1, maxFrac = 0.2 ){
    bb <- boundingBox( tracks )
    stepsByCell <- lapply(tracks, function(x){ subtracks(x,1) })
    atBorderAngle <- sapply( stepsByCell, angleCheck, bb, threshold = angleThreshold ) > maxFrac
    atBorderDistance <- sapply( stepsByCell, distanceCheck, bb, threshold = distanceThreshold ) > maxFrac
    atBorder <- atBorderAngle & atBorderDistance

    return( tracks[!atBorder] )
}
```

Now apply these to the datasets; plot the removed cells:

```
par( mfrow=c(1,3) )
old <- TCells
TCells <- notAtBorder( TCells )
TRemoved <- old[ !is.element( names(old), names(TCells) ) ]
plot( TRemoved, col = "red" )

old <- BCells
BCells <- notAtBorder( BCells )
BRemoved <- old[ !is.element( names(old), names(BCells) ) ]
plot( BRemoved, col = "red" )

old <- Neutrophils
Neutrophils <- notAtBorder( Neutrophils )
NRemoved <- old[ !is.element( names(old), names(Neutrophils) ) ]
plot( NRemoved, col = "red" )
```

```
# show how many removed:
c( paste0( "T cells : ", length( TRemoved), " of ", 
           length( TRemoved ) + length( TCells ), " tracks removed"),
   paste0( "B cells : ", length( BRemoved), " of ", 
           length( BRemoved ) + length( BCells ), " tracks removed"),
   paste0( "Neutrophils : ", length( NRemoved), " of ", 
           length( NRemoved ) + length( Neutrophils ), " tracks removed")
   )
```

```
## [1] "T cells : 13 of 258 tracks removed"    
## [2] "B cells : 4 of 97 tracks removed"      
## [3] "Neutrophils : 13 of 457 tracks removed"
```

Indeed, this seems to remove some cells at the X and Y borders – but not too many.

Now project to the XY plane to deal with the z-dimension as discussed earlier:

```
TCells <- projectDimensions( TCells, c("x","y") )
BCells <- projectDimensions( BCells, c("x","y") )
Neutrophils <- projectDimensions( Neutrophils, c("x","y") )
```

All border artifacts have now been dealt with.

## 4 Non-motile cells?

To remove non-motile cells, we apply the code from section 5.2 in the vignette on quality control methods to all datasets; in this case, we chose to remove cells that clearly are not moving so that example analyses in the package are actually based on a motile population.

Define the required functions (see the vignette on quality control methods):

```
bicNonMotile <- function( track, sigma ){
  
  # we'll use only x and y coordinates since we saw earlier that the z-dimension was
  # not so reliable
  allPoints <- track[,c("x","y")]
  
  # Compute the log likelihood under a multivariate gaussian.
  # For each point, we get the density under the Gaussian distribution 
  # (using dmvnorm from the mvtnorm package).
  # The product of these densities is then the likelihood; but since we need the 
  # log likelihood, we can also first log-transform and then sum:
  Lpoints <- mvtnorm::dmvnorm( allPoints, 
                      mean = colMeans(allPoints), # for a Gaussian around the mean position
                      sigma = sigma*diag(2), # sd of the Gaussian (which we should choose)
                      log = TRUE )
  logL <- sum( Lpoints )
  
  # BIC = k log n - 2 logL; here k = 3 ( mean x, mean y, sigma )
  return( 3*log(nrow(allPoints)) - 2*logL )
}
# the BIC for a given cutoff m
bicAtCutoff <- function( track, m, sigma ){
  
  # we'll use only x and y coordinates since we saw earlier that the z-dimension was
  # not so reliable
  allPoints <- track[,c("x","y")]
  
  # Split into two coordinate sets based on the cutoff m:
  firstCoords <- allPoints[1:m, , drop = FALSE]
  lastCoords <- allPoints[(m+1):nrow(allPoints), , drop = FALSE ]
  
  # Compute log likelihood under two separate Gaussians:
  Lpoints1 <- mvtnorm::dmvnorm( firstCoords, 
    mean = colMeans(firstCoords), 
    sigma = sigma*diag(2), 
    log = TRUE )
  Lpoints2 <- mvtnorm::dmvnorm( lastCoords, 
    mean = colMeans(lastCoords), 
    sigma = sigma*diag(2), 
    log = TRUE )
  logL <- sum( Lpoints1 ) + sum( Lpoints2 )
  
  # BIC = k log n - 2 logL; here k = 6 ( 2*mean x, 2*mean y, sigma, and m )
  return( 6*log(nrow(allPoints)) - 2*logL )
}

# We'll try all possible cutoffs m, and choose best model (minimal BIC)
# to compare to our non-motile "null hypothesis":
bicMotile <- function( track, sigma ){
  
  # cutoff anywhere from after the first two coordinates to 
  # before the last two (we want at least 2 points in each Gaussian,
  # to prevent fitting of a single point)
  cutoffOptions <- 2:(nrow(track)-2)
  
    min( sapply( cutoffOptions, function(m) bicAtCutoff(track,m,sigma) ) )
}

# Delta BIC between the two models
deltaBIC <- function( x, sigma ){
  b1 <- bicNonMotile( x, sigma )
  b2 <- bicMotile( x, sigma )
  d <- b1 - b2
  d
}
```

Now apply to the different datasets. We use sigma = 7 to focus on cells that move notably further than their own size:

```
TCellsBIC <- sapply( TCells, deltaBIC, 7 )
BCellsBIC <- sapply( BCells, deltaBIC, 7 )
NeutrophilsBIC <- sapply( Neutrophils, deltaBIC, 7 )

# Keep only the motile cells; BIC > 6 means reasonable evidence for motility
TNonMotile <- TCells[ TCellsBIC < 6 ]
BNonMotile <- BCells[ BCellsBIC < 6 ]
NNonMotile <- Neutrophils[ NeutrophilsBIC < 6 ]

TCells <- TCells[ TCellsBIC >= 6 ]
BCells <- BCells[ BCellsBIC >= 6 ]
Neutrophils <- Neutrophils[ NeutrophilsBIC >= 6 ]

# Check how many removed:
c( paste0( "T cells : ", length( TNonMotile), " of ", 
           length( TNonMotile ) + length( TCells ), " tracks removed"),
   paste0( "B cells : ", length( BNonMotile), " of ", 
           length( BNonMotile ) + length( BCells ), " tracks removed"),
   paste0( "Neutrophils : ", length( NNonMotile), " of ", 
           length( NNonMotile ) + length( Neutrophils ), " tracks removed")
   )
```

```
## [1] "T cells : 48 of 245 tracks removed"    
## [2] "B cells : 19 of 93 tracks removed"     
## [3] "Neutrophils : 73 of 444 tracks removed"
```

```
# Plot for comparison:
par(mfrow=c(3,2))
plot( TCells, main = "T cells (motile)" )
plot( TNonMotile, main = "T cells (non-motile)" )
plot( BCells, main = "B cells (motile)" )
plot( BNonMotile, main = "B cells (non-motile)" )
plot( Neutrophils, main = "Neutrophils (motile)" )
plot( NNonMotile, main = "Neutrophils (non-motile)" )
```

This filter seems sensible in the cells it removes, so we will keep the filtered “motile” datasets.

## 5 Double tracking?

We check for double tracking by considering cell pairs with less than a 10 degree angle between them and at a distance less than 10. There are no double-tracking errors in these datasets. (We will not run the code below to avoid clutter from many plots, but you can use it to check the absence of double-tracking errors for yourself).

```
checkPotentialDoubles <- function( tracks, distanceThreshold = 10, angleThreshold = 10 ){
  # na.omit because when cells do not share time points, their distance is NA.
  pairs <- na.omit( analyzeCellPairs( tracks ) )
  check <- pairs[ pairs$dist <= distanceThreshold & pairs$angle <= angleThreshold, ]
  
  # return if no pairs to check
  if( nrow(check) == 0 ){
    message("No suspicious pairs found!")
    return(NULL)
  }
  
  # Plot suspicious pairs; let user navigate with keystrokes:
  oldpar <- par()
  par( mfrow=c(2,2), mar=c(0, 0, 4, 0))
  for( i in 1:nrow( check ) ) {
    c1 <- pairs$cell1[i]; c2 <- pairs$cell2[i]
    plot( tracks[c(c1,c2)], main = paste0( c1,"-",c2),axes=FALSE, 
        frame.plot=TRUE, xlab=NA, ylab=NA )
    if( i %% 4 == 0 ) invisible(readline(prompt="Press [enter] to continue"))
  }
  par( oldpar )
  
  return(check)
}

checkPotentialDoubles( TCells )
checkPotentialDoubles( BCells )
checkPotentialDoubles( Neutrophils )
```

### 6.6 Gap correction

Another possible artifact is the existence of “gaps”: missing coordinates in a track. To detect this, we first check the time resolution of all the datasets:

```
# Check median dt for all datasets:
all.data <- list( TCells = TCells, BCells = BCells, Neutrophils = Neutrophils )
lapply( all.data, timeStep )
```

```
## $TCells
## [1] 24
## 
## $BCells
## [1] 24
## 
## $Neutrophils
## [1] 24
```

They all have the same median imaging frequency of 24 seconds between images. Now check all steps in each dataset for a range of step durations:

```
# Find durations of all steps in each dataset
step.dt <- lapply( all.data, function(x) {
  steps <- subtracks(x,1)
  sapply( steps, duration)
})

# Check the range of these durations:
range.dt <- lapply( step.dt, range )
range.dt
```

```
## $TCells
## [1] 24 48
## 
## $BCells
## [1] 24 48
## 
## $Neutrophils
## [1] 24 48
```

So while the median frequency is every 24 seconds, there are in all datasets also steps of 48 seconds. This suggests that some coordinates are missing. We need to correct for that, since such long steps may distort step-based statistics (like displacements and turning angles).

Check how bad the problem is:

```
percentage.missing <- lapply( step.dt, function(x) 100*sum( x != 24 ) / length(x) )
percentage.missing
```

```
## $TCells
## [1] 0.1536492
## 
## $BCells
## [1] 0.04636069
## 
## $Neutrophils
## [1] 1.681484
```

So not too many coordinates are missing. Still, to fix this, let’s use `repairGaps` with method “split”:

```
# Split tracks when there is a gap; after splitting, keep only tracks of at least length 4.
TCells <- repairGaps( TCells, how = "split", split.min.length = 4 )
BCells <- repairGaps( BCells, how = "split", split.min.length = 4 )
Neutrophils <- repairGaps( Neutrophils, how = "split", split.min.length = 4 )

# check that it has worked:
corrected.data <- list( TCells = TCells, BCells = BCells, Neutrophils = Neutrophils )
step.dt <- lapply( corrected.data, function(x) {
  steps <- subtracks(x,1)
  sapply( steps, duration)
})
lapply( step.dt, range )
```

```
## $TCells
## [1] 24 24
## 
## $BCells
## [1] 24 24
## 
## $Neutrophils
## [1] 24 24
```

Now, all coordinates are evenly spaced.

### 6.7 Time resolution

Since we already saw in the previous section that the three datasets have the same imaging frequency, we do not need to correct for this in any downstream analysis. Thus, these are the datasets we will use in the rest of the package.
