# Supplementary material for: CelltrackR: An R package for fast and flexible analysis of immune cell migration data
Source: Immunoinformatics (Amst). Author manuscript; Available in PMC 2023 Apr 6. (PMC10079262; doi:10.1016/j.immuno.2021.100003)
Supplement: Supplemental movies and figures [file NIHMS1884788-supplement-Supplemental_movies_and_figures.zip › SF4-ana-methods.html]

Track Analysis Methods


# Track Analysis Methods

#### Inge Wortel

#### 2021-07-07

# Introduction

This tutorial provides an overview of how to perform popular analysis methods and visualizations commonly applied to tracking data. Examples range from the simple analysis of speed or other measures on single tracks, to the application of step-based or staggered metrics, to the generation of autocorrelation plots and performing angle analyses.

# Datasets

First load the package:

```
library( celltrackR )
library( ggplot2 )
```

The package contains a dataset of B and T cells in a mouse cervical lymph node, and neutrophils responding to an *S.aureus* infection in a mouse ear, all imaged using two-photon microscopy. While the original data contained 3D coordinates, we’ll use the 2D projection on the XY plane (see the vignettes on quality control methods and preprocessing of the package datasets for details). We will here use this dataset as an example of how to apply analysis metrics.

For example, the T-cell dataset consists of 199 tracks of individual cells in a tracks object:

```
str( TCells, list.len = 1 )
```

```
## List of 199
##  $ 1     : num [1:39, 1:3] 48 72 96 120 144 168 192 216 240 264 ...
##   ..- attr(*, "dimnames")=List of 2
##   .. ..$ : NULL
##   .. .. [list output truncated]
##   [list output truncated]
##  - attr(*, "class")= chr "tracks"
```

Each element in this list is a track from a single cell, consisting of a matrix with \((x,y)\) coordinates and the corresponding measurement timepoints:

```
head( TCells[[1]] )
```

```
##        t       x       y
## [1,]  48 90.8534 65.3943
## [2,]  72 89.5923 64.9042
## [3,]  96 88.6958 67.1125
## [4,] 120 87.3437 68.2392
## [5,] 144 86.2740 67.9236
## [6,] 168 84.0549 68.2502
```

Similarly, we will also use the `BCells` and `Neutrophils` data:

```
str( BCells, list.len = 1 )
```

```
## List of 74
##  $ 130  : num [1:24, 1:3] 48 72 96 120 144 168 192 216 240 264 ...
##   ..- attr(*, "dimnames")=List of 2
##   .. ..$ : NULL
##   .. .. [list output truncated]
##   [list output truncated]
##  - attr(*, "class")= chr "tracks"
```

```
str( Neutrophils, list.len = 1 )
```

```
## List of 411
##  $ 21     : num [1:7, 1:3] 48 72 96 120 144 ...
##   ..- attr(*, "dimnames")=List of 2
##   .. ..$ : NULL
##   .. .. [list output truncated]
##   [list output truncated]
##  - attr(*, "class")= chr "tracks"
```

For the purpose of this tutorial, we’ll just sample a subset from these datasets (Normally you would not do this, but it will help here to speed things up and to avoid cluttered plots; the same methods would apply to a full dataset).

```
# for reproducibility
set.seed(2021)

# sample names, sort them in numeric order, and use them to subset the tracks
Bsample <- sample( names(BCells),50)
BCells2 <- BCells[ Bsample ]

# same for the T cells
Tsample <- sample( names(TCells),50)
TCells2 <- TCells[ Tsample ]

# and the neutrophils
Nsample <- sample( names(Neutrophils),50)
Neutrophils2 <- Neutrophils[ Nsample ]
```

# 1 Simple track visualization

### 1.1 2D and 3D plotting

The standard plotting method for tracks plots the projection of tracks on the \((x,y)\) plane (if tracks are in 3D, the z-coordinate is ignored to obtain a projection):

```
par( mfrow = c(1,3), mar = c(2,2,3,1)+0.1 )
plot( TCells2, main = "T cells" )
plot( BCells2, main = "B cells" )
plot( Neutrophils2, main = "Neutrophils" )
```

We can also plot 3D tracks using the function `plot3d()` (which requires the package `scatterplot3d`):

```
# load original, 3D tracks as an example, since the processed tracks are 2D
load( system.file("extdata", "TCellsRaw.rda", package="celltrackR" ) )
load( system.file("extdata", "BCellsRaw.rda", package="celltrackR" ) )
load( system.file("extdata", "NeutrophilsRaw.rda", package="celltrackR" ) )

# omit axes and labels to save space here;
par( mfrow = c(1,3), mar = c(0,0,2,0) + 0.1 )
plot3d( TCellsRaw, main = "T cells", tick.marks = FALSE )
plot3d( BCellsRaw, main = "B cells", tick.marks = FALSE )
plot3d( NeutrophilsRaw, main = "Neutrophils", tick.marks = FALSE )
```

### 1.2 Star plots (Rose plots)

Finally, we can overlay the track starting points using `normalizeTracks()` – which is useful to detect directionality:

```
par( mfrow = c(1,3), mar = c(2,2,3,1)+0.1 )
plot( normalizeTracks(TCells2), main = "T cells" )
plot( normalizeTracks(BCells2), main = "B cells" )
plot( normalizeTracks(Neutrophils2), main = "Neutrophils" )
```

# 2 Quantification of measures on tracks

The package contains several measures that can be computed on single tracks (see `?TrackMeasures` for an overview). The following sections will give examples on how to compute these metrics in different ways, using the `speed()` function as an example.

### 2.1 Cell-based analyses

To compute a metric for each track in a dataset, use R’s `sapply()` function directly on the tracks object:

```
# Obtain mean speeds for each track using sapply
Tcell.speeds <- sapply( TCells2, speed )
str( Tcell.speeds )
```

```
##  Named num [1:50] 0.0804 0.2038 0.0873 0.2824 0.1394 ...
##  - attr(*, "names")= chr [1:50] "25" "5828_2" "8793" "6782" ...
```

```
summary( Tcell.speeds )
```

```
##    Min. 1st Qu.  Median    Mean 3rd Qu.    Max. 
## 0.04102 0.09340 0.13911 0.14266 0.19052 0.28240
```

This returns a mean speed for each of the 199 tracks in the `TCell` dataset, which can be analyzed further or plotted using any of R’s default methods, e.g.:

```
hist( Tcell.speeds )
```

Now let’s repeat for the other two celltypes and plot them next to each other for comparison

```
Bcell.speeds <- sapply( BCells2, speed )
Nphil.speeds <- sapply( Neutrophils2, speed )

# Create a dataframe of all data
dT <- data.frame( cells = "T cells", speed = Tcell.speeds )
dB <- data.frame( cells = "B cells", speed = Bcell.speeds )
dN <- data.frame( cells = "Neutrophils", speed = Nphil.speeds )
d <- rbind( dT, dB, dN )

# Compare:
ggplot( d, aes( x = cells, y = speed ) ) +
  ggbeeswarm::geom_quasirandom() +
  scale_y_continuous( limits = c(0,NA) ) +
  theme_classic()
```

### 2.2 Step-based analyses

It has been suggested that cell-based analyses introduce biases, and that analyses should be performed on individual “steps” from all tracks combined instead (Beltman et al (2009)).

We can extract such steps using the `subtracks()` function (where `i` is the subtrack length, which is 1 for a single step):

```
Tcell.steps <- subtracks( TCells2, i = 1 )
```

We can then proceed as before to obtain speeds for each step and further summary statistics:

```
# Obtain instantaneous speeds for each step using sapply
Tcell.step.speeds <- sapply( Tcell.steps, speed )
str( Tcell.step.speeds )
```

```
##  Named num [1:988] 0.1364 0.1223 0.057 0.2002 0.0684 ...
##  - attr(*, "names")= chr [1:988] "25.1" "25.2" "25.3" "25.4" ...
```

```
summary( Tcell.step.speeds )
```

```
##     Min.  1st Qu.   Median     Mean  3rd Qu.     Max. 
## 0.002167 0.047966 0.090478 0.127717 0.160476 0.868418
```

Compare cell-based versus step-based:

```
d.step <- data.frame( method = "step-based", speed = Tcell.step.speeds )
d.cell <- data.frame( method = "cell-based", speed = Tcell.speeds )
d.method <- rbind( d.step, d.cell )

ggplot( d.method, aes( x = method, y = speed ) ) +
  ggbeeswarm::geom_quasirandom( size = 0.3 ) +
  scale_y_continuous( limits = c(0,NA) ) +
  theme_classic()
```

And again compare between the different celltypes:

```
Bcell.step.speeds <- sapply( subtracks(BCells2,1), speed )
Nphil.step.speeds <- sapply( subtracks(Neutrophils2,1), speed )

# Create a dataframe of all data
dT <- data.frame( cells = "T cells", speed = Tcell.step.speeds )
dB <- data.frame( cells = "B cells", speed = Bcell.step.speeds )
dN <- data.frame( cells = "Neutrophils", speed = Nphil.step.speeds )
dstep <- rbind( dT, dB, dN )

# Compare (violin plot now because there are many individual steps)
ggplot( dstep, aes( x = cells, y = speed ) ) +
  geom_violin() +
  #ggbeeswarm::geom_quasirandom( size = 0.3 ) +
  scale_y_continuous( limits = c(0,NA) ) +
  theme_classic()
```

Note that we can do something similar for turning angles, but we need at least two steps to compute a turning angle. Therefore, use `subtracks()` with `i=2` in combination with the `overallAngle()` method:

```
Tcell.step.angle <- sapply( subtracks(TCells2,2), overallAngle )
Bcell.step.angle <- sapply( subtracks(BCells2,2), overallAngle )
Nphil.step.angle <- sapply( subtracks(Neutrophils2,2), overallAngle )

# Create a dataframe of all data
dT <- data.frame( cells = "T cells", turn.angle = Tcell.step.angle )
dB <- data.frame( cells = "B cells", turn.angle = Bcell.step.angle )
dN <- data.frame( cells = "Neutrophils", turn.angle = Nphil.step.angle )
dangle <- rbind( dT, dB, dN )

# convert radians to degrees
dangle$turn.angle <- pracma::rad2deg( dangle$turn.angle )

# Compare:
ggplot( dangle, aes( x = cells, y = turn.angle ) ) +
  #ggbeeswarm::geom_quasirandom( size = 0.3 ) +
  geom_violin() +
  scale_y_continuous( limits = c(0,NA) ) +
  theme_classic()
```

### 2.3 Staggered analyses

Another approach is to compute metrics not only on “steps” (subtracks of length one), but on all possible subtracks in a track. This is called a “staggered” analysis (Mohktari et al (2013)).

To see how this works on a single track, use `applyStaggered` with `matrix=TRUE`. For example, consider track number 4 (id = “6782”) in the `TCells2` data, which has 8 coordinates (7 steps):

```
x <- TCells2[[4]]
x
```

```
##        t       x       y
## [1,] 288 238.755 143.881
## [2,] 312 238.844 130.222
## [3,] 336 242.063 143.037
## [4,] 360 244.999 145.920
## [5,] 384 245.726 148.873
## [6,] 408 246.592 151.434
## [7,] 432 249.139 151.902
## [8,] 456 257.207 150.965
```

If we compute the speed on all staggered subtracks, we get the following matrix:

```
options(width = 120)
stag <- applyStaggered( x, speed, matrix = TRUE )
stag
```

```
##           [,1]      [,2]      [,3]      [,4]      [,5]      [,6]      [,7]      [,8]
## [1,]        NA 0.5691371 0.5598416 0.4303781 0.3544625 0.3060988 0.2730659 0.2824031
## [2,] 0.5691371        NA 0.5505461 0.3609986 0.2829042 0.2403392 0.2138517 0.2346141
## [3,] 0.5598416 0.5505461        NA 0.1714510 0.1490833 0.1369369 0.1296781 0.1714277
## [4,] 0.4303781 0.3609986 0.1714510        NA 0.1267156 0.1196798 0.1157537 0.1714219
## [5,] 0.3544625 0.2829042 0.1490833 0.1267156        NA 0.1126440 0.1102728 0.1863239
## [6,] 0.3060988 0.2403392 0.1369369 0.1196798 0.1126440        NA 0.1079016 0.2231639
## [7,] 0.2730659 0.2138517 0.1296781 0.1157537 0.1102728 0.1079016        NA 0.3384262
## [8,] 0.2824031 0.2346141 0.1714277 0.1714219 0.1863239 0.2231639 0.3384262        NA
```

Where point \((i,j)\) contains the mean speed in the subtrack from the \(i^{th}\) to the \(j^{th}\) coordinate (thus, the diagonal contains subtracks from a coordinate to itself – which is no subtrack at all but a single coordinate, and therefore has no speed). Visualize this using the `image()` or `filled.contour()` functions:

```
image( stag )
```

```
filled.contour( stag )
```

Now do it again for a longer track:

```
filled.contour( applyStaggered( TCells2[[1]], speed, matrix = TRUE ) )
```

This nicely illustrates fluctuations in speed at different points in the track.

If we leave out the `matrix=TRUE`, the function simply returns the mean of all non-NA entries in the matrix:

```
# These are the same:
applyStaggered( TCells2[[1]], speed )
```

```
## [1] 0.07896278
```

```
mean( stag, na.rm = TRUE )
```

```
## [1] 0.2510543
```

`applyStaggered` only works on a single track, not a tracks object. To directly get the staggered means for all tracks in the object, use the `staggered` option:

```
# Compare the three different methods
Tcell.stag.speed <- sapply( TCells2, staggered( speed ) )
d.staggered <- data.frame( method = "staggered", speed = Tcell.speeds )
d.method <- rbind( d.step, d.cell, d.staggered )

ggplot( d.method, aes( x = method, y = speed ) ) +
  ggbeeswarm::geom_quasirandom( size = 0.3 ) +
  scale_y_continuous( limits = c(0,NA) ) +
  theme_classic()
```

```
# cell-based and staggered cell-based are slightly different:
summary( Tcell.speeds - Tcell.stag.speed )
```

```
##      Min.   1st Qu.    Median      Mean   3rd Qu.      Max. 
## -0.040826 -0.002639  0.002228  0.004195  0.009487  0.052823
```

# 3 Mean square displacement plots

A common analysis on migration data is generating mean square displacement (MSD) plots. For a range of time interval \(\Delta t\), we compute the average squared displacement of all subtracks with that duration. Like with step-based approaches, we compute the metric on subtracks pooled from all individual cells – but now we also do this for subtracks of more than one step.

We can use the `subtracks()` function with varying `i` argument to obtain subtracks of different lengths, and then use `sapply()` to find a mean metric for each of these datasets. However, the package also contains a convenience function that does this automatically: `aggregate()`. For example, use this function to directly get the mean square displacement of all single steps:

```
aggregate( TCells2, squareDisplacement, subtrack.length = 1 )
```

```
##   i    value
## 1 1 19.26447
```

where “i” is the subtrack length in number of steps, and “value” is the average value of the applied metric (in this case, square displacement) for all subtracks of that length in the data.

We can also report different summary statistics by changing the `FUN` argument (see `?aggregate.tracks`):

```
aggregate( TCells2, squareDisplacement, subtrack.length = 1, FUN = "mean.se" )
```

```
##   i     mean    lower    upper
## 1 1 19.26447 17.65995 20.86899
```

This gives us not only the mean, but also the standard error.

Now, if we leave out the `subtrack.length`, `aggregate()` automatically computes the metric for subtracks of all possible lengths in the dataset and returns those in a dataframe:

```
Tcell.msd <- aggregate( TCells2, squareDisplacement, FUN = "mean.se" )
str( Tcell.msd )
```

```
## 'data.frame':    39 obs. of  4 variables:
##  $ i    : num  1 2 3 4 5 6 7 8 9 10 ...
##  $ mean : num  19.3 41.5 67.9 97.1 128.3 ...
##  $ lower: num  17.7 39 64.4 92.4 122 ...
##  $ upper: num  20.9 43.9 71.3 101.9 134.5 ...
```

We can then use this for plotting:

```
ggplot( Tcell.msd, aes( x = i, y = mean ) ) +
  geom_ribbon( aes( ymin = lower, ymax = upper) , alpha = 0.2 ,color=NA ) +
  geom_line( ) +
  labs( x = expression( paste(Delta,"t (steps)") ),
        y = "mean square displacement") +
  theme_classic()
```

Plotting the standard error is useful because, as \(\Delta t\) increases, the number of subtracks with that length decreases rapidly. MSD values at the high end of the plot therefore tend to be less reliable as they are based on very few observations.

Compare for example the number of subtracks of length 10 to the number of length 35:

```
num10 <- length( subtracks( TCells2, 10 ) )
num35 <- length( subtracks( TCells2, 35 ) )
c( "10" = num10, "35" = num35 )
```

```
##  10  35 
## 562  46
```

Now, let’s compare the MSD plot for the three different types of cells:

```
# Combine into a single dataframe with one column indicating the celltype
# To truly compare them, report subtrack length not in number of steps but
# in their duration (which may differ between different datasets)
Tcell.msd$cells <- "T cells"
Tcell.msd$dt <- Tcell.msd$i * timeStep( TCells2 )
Bcell.msd <- aggregate( BCells2, squareDisplacement, FUN = "mean.se" )
Bcell.msd$cells <- "B cells"
Bcell.msd$dt <- Bcell.msd$i * timeStep( BCells2 )
Nphil.msd <- aggregate( Neutrophils2, squareDisplacement, FUN = "mean.se" )
Nphil.msd$cells <- "Neutrophils"
Nphil.msd$dt <- Nphil.msd$i * timeStep( Neutrophils2 )
msddata <- rbind( Tcell.msd, Bcell.msd, Nphil.msd )
head(msddata)
```

```
##   i      mean     lower     upper   cells  dt
## 1 1  19.26447  17.65995  20.86899 T cells  24
## 2 2  41.45140  38.97172  43.93109 T cells  48
## 3 3  67.85251  64.43261  71.27241 T cells  72
## 4 4  97.12745  92.37496 101.87993 T cells  96
## 5 5 128.25329 121.99665 134.50993 T cells 120
## 6 6 157.86364 149.92798 165.79929 T cells 144
```

```
# Plot
p1 <- ggplot( msddata, aes( x = dt , y = mean, color = cells, fill = cells ) ) +
  geom_ribbon( aes( ymin = lower, ymax = upper) , alpha = 0.2 ,color=NA ) +
  geom_line( ) +
  labs( x = expression( paste(Delta,"t (seconds)") ),
        y = "mean square displacement") +
  theme_classic() + theme(
    legend.position = "top"
  )
# Also make a zoomed version to look only at first part of the plot
pzoom <- p1 + coord_cartesian( xlim = c(0,500), ylim = c(0,3000) ) 
gridExtra::grid.arrange( p1, pzoom, ncol = 2 )
```

Note here that the bigger \(\Delta t\), the less reliable the MSD becomes. This is especially the case when the imaging window is limited (as is often the case for two-photon experiments). The longer \(\Delta t\), the more cells have the chance to leave the imaging window during that time, which means that there will be less data available to compute a mean on. Moreover, the data that is still there is biased: faster and more persistent cells will leave the imaging window more quickly, and are therefore underrepresented in the displacements measured at large \(\Delta t\). This, for example, is one reason why the T-cell curve seems to flatten at larger \(\Delta t\).

> Tip: `aggregate()` can also make MSD plots from non-overlapping subtracks or from subtracks starting at the first position of each track; see `?aggregate.tracks` for details.

# 4 Analyzing persistence

The downside of the MSD plot is that it is strongly affected by both speed *and* directionality of cells: faster cells displace more, as do cells that move straighter. It can therefore be worthwile to study cell speed and straightness or “persistence” independently.

### 4.1 Straightness metrics

The package implements several metrics that provide a measure of track straightness or directionality (see Mohktari et al (2013) for details): `displacementRatio()`, `outreachRatio`, `straightness()`, and `asphericity()`. These metrics can be applied to the data in the same way as was done for `speed()` in section 2 of this tutorial. See also `?TrackMeasures` for more information.

### 4.2 Autocorrelation plots

To analyze cell persistence, another good analysis is the autocorrelation plot: taking steps with \(\Delta t\) time in between, how much do their directions of movement still correlate?

Officially, the autocorrelation plot is a plot of \(\cos \alpha\) of two steps, \(\vec{s\_1}\) and \(\vec{s\_2}\), whose starting points are \(\Delta t\) apart. This is equal to the dot product between \(\vec{s\_1}\) and \(\vec{s\_2}\) after normalizing both vectors to unit length. We can compute this again using `aggregate()` (see also the previous section):

```
Tcell.acor <- aggregate( TCells2, overallNormDot, FUN = "mean.se"  )
Tcell.acor$dt <- Tcell.acor$i * timeStep(TCells2)
Tcell.acor$cells <- "T cells"

ggplot( Tcell.acor, aes( x = dt , y = mean, color = cells, fill = cells ) ) +
  geom_hline( yintercept = 0 ) +
  geom_ribbon( aes( ymin = lower, ymax = upper) , alpha = 0.2 ,color=NA ) +
  geom_line( ) +
  labs( x = expression( paste(Delta,"t (seconds)") ),
        y = expression(cos(alpha))) +
  theme_classic() + 
  theme( axis.line.x = element_blank() )
```

Note, however, that steps in which the cell barely moves can add a lot of noise to the analysis of angles. Since it makes no sense anyway to speak of persistent movement when a cell is not moving, we can avoid this by filtering for pairs of steps \(\vec{s\_1}\) and \(\vec{s\_2}\) that have at least some minimum displacement. To do this, we give `aggregate()` a function in the `filter.subtracks` argument:

```
# Returns TRUE if first and last step of a track are of the minimum length of 1 micron
check <- function(x){ 
  all( sapply( list(head(x,2),tail(x,2)), trackLength ) >= 1.0 )
}
# repeat analysis
Tcell.acor2 <- aggregate( TCells2, overallNormDot, 
                     FUN = "mean.se", filter.subtracks = check )
Tcell.acor2$dt <- Tcell.acor2$i * timeStep(TCells2)
Tcell.acor2$cells <- "T cells (filtered)"

d <- rbind( Tcell.acor, Tcell.acor2 )

# compare
ggplot( d, aes( x = dt , y = mean, color = cells, fill = cells ) ) +
  geom_hline( yintercept = 0 ) +
  geom_line( ) +
  labs( x = expression( paste(Delta,"t (seconds)") ),
        y = expression(cos(alpha))) +
  theme_classic() + 
  theme( axis.line.x = element_blank() )
```

The drop in autocorrelation is now slightly less steep, because the lack of correlation between steps where the cell does not move is not taken into account.

Another solution is to make the autocovariance plot instead of the autocorrelation plot, where we plot the dot product between the original unnormalized vectors \(\vec{s\_1} \bullet \vec{s\_2}\) instead of normalizing them fist. The dot product is less sensitive to noise from very short steps and is therefore recommended:

```
# autocovariance
Tcell.acov <- aggregate( TCells2, overallDot, 
                     FUN = "mean.se", filter.subtracks = check )
Tcell.acov$dt <- Tcell.acov$i * timeStep(TCells2)
Tcell.acov$cells <- "T cells"


# compare
ggplot( Tcell.acov, aes( x = dt , y = mean, color = cells, fill = cells ) ) +
  geom_hline( yintercept = 0 ) +
  geom_line( ) +
  labs( x = expression( paste(Delta,"t (seconds)") ),
        y = "autocovariance" ) +
  theme_classic() + 
  theme( axis.line.x = element_blank() )
```

However, the autocovariance is no longer a number between 0 and 1, which is somewhat less convenient when comparing cell types moving at different speeds. We therefore divide all values by the maximum autocovariance at \(\Delta t = 0\), and compare with the other celltypes:

```
# Normalized autocovariance
Tcell.acov[,2:4] <- Tcell.acov[,2:4] / Tcell.acov$mean[1]

# Other cells
Bcell.acov <- aggregate( BCells2, overallDot, FUN = "mean.se" )
Bcell.acov$cells <- "B cells"
Bcell.acov$dt <- Bcell.acov$i * timeStep( BCells2 )
Bcell.acov[,2:4] <- Bcell.acov[,2:4] / Bcell.acov$mean[1]

Nphil.acov <- aggregate( Neutrophils2, overallDot, FUN = "mean.se" )
Nphil.acov$cells <- "Neutrophils"
Nphil.acov$dt <- Nphil.acov$i * timeStep( Neutrophils2 )
Nphil.acov[,2:4] <- Nphil.acov[,2:4] / Nphil.acov$mean[1]

acovdata <- rbind( Tcell.acov, Bcell.acov, Nphil.acov )

# compare;
p1 <- ggplot( acovdata, aes( x = dt , y = mean, color = cells, fill = cells ) ) +
  geom_hline( yintercept = 0 ) +
  geom_ribbon( aes( ymin = lower, ymax = upper) , alpha = 0.2 ,color=NA ) +
  geom_line( ) +
  labs( x = expression( paste(Delta,"t (seconds)") ),
        y = "autocovariance" ) +
  theme_classic() + 
  theme( axis.line.x = element_blank(), 
         legend.position = "top" )

pzoom <- p1 + coord_cartesian( xlim = c(0,500), ylim  = c(-0.1,1) )
gridExtra::grid.arrange( p1, pzoom, ncol = 2 )
```

Note that neutrophils have a rapid initial drop in autocorrelation, but then maintain directional persistence over longer timescales; this is an indication of global directionality in the data (see below). T cells have a similar initial drop, but without the long-term directionality.

# 5 Analyzing directionality

Aside from directionality *within* a track segment (because of directional persistence), there can also be global directionality *between* different tracks: for example, when cells are moving in the direction of some target. The package supports several types of analyses to detect such directionality in a dataset.

### 5.1 Hotelling’s test

An unbiased method for detecting global directionality is Hotelling’s T-square test (see Textor et al (2011)), which tests if the mean step displacement vector is significantly different from the null vector. This method is powerful for detecting global movement biases when the direction is not known in advance.

For example, applying Hotelling’s test to the `Neutrophil` data:

```
par( mfrow=c(1,2) )
plot( Neutrophils2 )
hotellingsTest( Neutrophils2, plot = TRUE, col = "gray", step.spacing = 10 )
```

```
## 
##  Hotelling's one sample T2-test
## 
## data:  
## T2 = 25.489, df1 = 2, df2 = 78, p-value = 1.836e-05
## alternative hypothesis: true location is not equal to c(0,0)
```

This shows there is a bias towards movement in the \((1,-1)\) direction. See the discussion of tissue drift in the vignette on quality control for a more extensive discussion on how to use Hotelling’s test to detect a global directional bias.

### 5.2 Angle analyses

#### 5.2.1 Overview: Angles and distances of steps to a fixed reference (point, plane, or direction)

The following functions support the computation of angles and distances of steps compared to a fixed reference:

- `angleToPoint( x, from = 1, p = c(1,1,1) )` : angle to the reference point with coordinates `p`.
- `angleToDir( x, from = 1, dvec = c(1,1,1) )` : angle to a reference direction specified by vector `dvec`.
- `angleToPlane( x, from = 1, p1, p2, p3 )` : angle to a plane specified by the points `p1`, `p2`, and `p3`.
- `distanceToPlane( x, from = 1, p1, p2, p3 )` : distance to a plane specified by the points `p1`, `p2`, and `p3`.
- `distanceToPoint( x, from = 1, p )` : distance to the reference point with coordinates `p`.

These functions take each track in tracks object `x`, extract the single steps starting at index `from` (by default, this is the first step in the track), and compute the angle of this step towards the reference. The distance functions compute the distance of the step starting point to the reference.

#### 5.2.2 Angles and distances to planes: detecting tracking artefacts

Angles and distances in reference to a plane are useful to detect tracking artefacts, see the vignette on quality control for a detailed example.

#### 5.2.3 Angle to a reference direction: powerful directionality tests when direction is known

While Hotelling’s test may be used to test for directionality in an unknown direction, we can increase the power of testing when the direction is known (for example, in an experiment with an artificial chemotactic gradient in a known direction).

As an example, let’s make an artificial dataset with additional movement in the direction \((1,1)\):

```
# Directional movement is 0.05 micron/sec in each dimension
directional.speed <- c( 0.05, 0.05 )
add.dir <- function( x, speed.vector )
{
  # separate timepoints and coordinates
  tvec <- x[,1]
  coords <- x[,-1]
  
  # compute movement due to drift.
  speed.matrix <- matrix( rep( speed.vector, nrow(coords) ),
                          ncol = ncol(coords), byrow= TRUE )
  speed.matrix <- speed.matrix * tvec
  
  # Add drift to coordinates
  x[,-1] <- coords + speed.matrix
  return(x)
}

# Create data with directionality
TCells2.dir <- as.tracks( lapply( TCells2, add.dir, directional.speed ) )

# Plot both for comparison
# par(mfrow=c(1,2) )
# plot(TCells2, main = "original data" )
# plot(TCells2.dir, main = "with directional bias" )
```

Now, let’s view the distribution of step angles with the reference direction, and compare this to the original data:

```
step.angles <- sapply( subtracks( TCells2.dir, 1), angleToDir, dvec = c(1,1) )
step.angles.original <- sapply( subtracks( TCells2, 1 ), angleToDir, dvec = c(1,1) )
par(mfrow=c(1,2) )
hist( step.angles.original, main = "original data" )
hist( step.angles, main = "with directional bias" )
```

In the original dataset, angles to the reference direction form a sine distribution with a mean of 90 degrees – which is what we expect for 3D angles with a reference when there is no directional bias. But in the artificial dataset, there is an enrichment for angles below 90 degrees, indicative of a directional bias.

#### 5.2.4 Angles and distances to reference point: detecting movement towards a single point

Sometimes a directional bias is not in a fixed direction, but towards a fixed point. The direction of the bias then depends on the location a cell is in.

As an extreme example, let’s make an artificial dataset by normalizing all T cell tracks so that they end at the same endpoint:

```
# Normalize the endpoint by subtracting its coordinates from all other coordinates:
normalize.endpoint <- function( x ){
  coords <- x[,-1]
  coords.norm <- t( apply( coords, 1, function(a) a - coords[nrow(coords),] ) )
  x[,-1] <- coords.norm
  return(x)
}
TCells2.point <- as.tracks( lapply( TCells2, normalize.endpoint ) )

# Plot for comparison:
plot( TCells2.point, main = "with point attraction" )
```

Hotelling’s test cannot detect this bias, because the direction of the bias depends on where the track started:

```
hotellingsTest( TCells2.point, step.spacing = 10, col = "gray" )
```

```
## 
##  Hotelling's one sample T2-test
## 
## data:  
## T2 = 1.8532, df1 = 2, df2 = 109, p-value = 0.4023
## alternative hypothesis: true location is not equal to c(0,0)
```

Now, given that we know in which direction we expect the cells to move (towards point \((0,0)\)), we can again use angle analysis to compute the step angles towards the central point:

```
step.angles <- sapply( subtracks( TCells2.point, 1), angleToPoint, p = c(0,0) )
step.angles.original <- sapply( subtracks( TCells2, 1 ), angleToPoint, p = c(0,0) )
par(mfrow=c(1,2) )
hist( step.angles.original, main = "original data" )
hist( step.angles, main = "with point attraction" )
```

Once again, there is an significant enrichment for small angles.

We can also plot the angle versus the distance to the point:

```
step.distances <- sapply( subtracks( TCells2.point, 1), distanceToPoint, p = c(0,0) )

df <- data.frame( dist = step.distances,
                  angles = step.angles )
ggplot( df, aes( x = dist, y = angles ) ) +
  geom_point( size = 0.2, color = "gray") +
  geom_hline( yintercept = 90, color = "black" ) +
  stat_smooth( color = "red", method = "loess" ) +
  labs( x = "distance to reference point",
        y = "angle to reference point" ) +
  theme_classic()
```

```
## `geom_smooth()` using formula 'y ~ x'
```

In our artificial dataset, the average angle is below 90 degrees for any distance. But in case of true attraction towards a given point, we might expect the attraction to decrease as the distance to the point increases (for example, because cells at large distances do not feel the chemotactic gradient as much).

#### 5.2.5 Angles and distances between pairs of cells or individual steps

Analyze angles and distances between pairs of cells. Because computing all pairs of cells is slow, we’ll here take a sample of the tracks to speed things up a little

```
df.cells <- analyzeCellPairs( TCells2 )

# Plot
ggplot( df.cells, aes( x = dist, y = angle ) ) +
  geom_point( color = "gray" ) +
  stat_smooth( span = 1, color = "red" )+
  labs( x = "distance between cell pairs",
        y = "angle between cell pairs" ) +
  geom_hline( yintercept = 90, color = "black" ) +
  theme_classic()
```

```
## `geom_smooth()` using method = 'gam' and formula 'y ~ s(x, bs = "cs")'
```

```
## Warning: Removed 249 rows containing non-finite values (stat_smooth).
```

```
## Warning: Removed 249 rows containing missing values (geom_point).
```

We expect angles between cell pairs to be on average 90 degrees. A lower average angle for cell pairs at low distances may indicate the formation of cell streams and collective migration. A systematic deviation from the 90 degree mean may indicate global directionality in the data.

We can do something similar on the level of single steps (where we take into account only pairs of steps that occur at the same time point, and we filter for steps that displace at least two micron):

```
df.steps <- analyzeStepPairs( TCells2, filter.steps = function(x) displacement(x)>2  )

# Plot
ggplot( df.steps, aes( x = dist, y = angle ) ) +
  geom_point( color = "gray", size = 0.5 ) +
  stat_smooth( color = "red" )+
  labs( x = "distance between step pairs",
        y = "angle between step pairs") +
  geom_hline( yintercept = 90, color = "black" ) +
  theme_classic()
```

Again, we expect to see an average angle of 90 degrees for all distances. See also the vignette on quality control.
