# Supplementary material for: CelltrackR: An R package for fast and flexible analysis of immune cell migration data
Source: Immunoinformatics (Amst). Author manuscript; Available in PMC 2023 Apr 6. (PMC10079262; doi:10.1016/j.immuno.2021.100003)
Supplement: Supplemental movies and figures [file NIHMS1884788-supplement-Supplemental_movies_and_figures.zip › SF5-clustering.html]

Clustering Tracks with CelltrackR


# Clustering Tracks with CelltrackR

#### Inge Wortel

#### 2021-07-07

# Introduction

To group tracks with similar properties, one can in principle perform any clustering method of interest on a *feature matrix* of quantification metrics for each track in the dataset. The package comes with three convenience functions – `getFeatureMatrix()`,`trackFeatureMap()`, and `clusterTracks()` – to easily compute several metrics on all tracks at once, visualize them in 2 dimensions, and to cluster tracks accordingly. This tutorial shows how to use these functions to explore heterogeneity in a track dataset.

# Datasets

First load the package:

```
library( celltrackR )
```

The package contains a dataset of B and T cells in a mouse cervical lymph node, and neutrophils responding to an *S. aureus* infection in a mouse ear; all are imaged using two-photon microscopy. While the original data contained 3D coordinates, we’ll use the 2D projection on the XY plane (see the vignettes on quality control methods and preprocessing of the package datasets for details).

The T-cell dataset consists of 199 tracks of individual cells in a tracks object:

```
str( TCells, list.len = 2 )
#> List of 199
#>  $ 1     : num [1:39, 1:3] 48 72 96 120 144 168 192 216 240 264 ...
#>   ..- attr(*, "dimnames")=List of 2
#>   .. ..$ : NULL
#>   .. ..$ : chr [1:3] "t" "x" "y"
#>  $ 3     : num [1:19, 1:3] 24 48 72 96 120 144 168 192 216 240 ...
#>   ..- attr(*, "dimnames")=List of 2
#>   .. ..$ : NULL
#>   .. ..$ : chr [1:3] "t" "x" "y"
#>   [list output truncated]
#>  - attr(*, "class")= chr "tracks"
```

Each element in this list is a track from a single cell, consisting of a matrix with \((x,y)\) coordinates and the corresponding measurement timepoints:

```
head( TCells[[1]] )
#>        t       x       y
#> [1,]  48 90.8534 65.3943
#> [2,]  72 89.5923 64.9042
#> [3,]  96 88.6958 67.1125
#> [4,] 120 87.3437 68.2392
#> [5,] 144 86.2740 67.9236
#> [6,] 168 84.0549 68.2502
```

Similarly, we will also use the `BCells` and `Neutrophils` data:

```
str( BCells, list.len = 2 )
#> List of 74
#>  $ 130  : num [1:24, 1:3] 48 72 96 120 144 168 192 216 240 264 ...
#>   ..- attr(*, "dimnames")=List of 2
#>   .. ..$ : NULL
#>   .. ..$ : chr [1:3] "t" "x" "y"
#>  $ 210  : num [1:39, 1:3] 48 72 96 120 144 168 192 216 240 264 ...
#>   ..- attr(*, "dimnames")=List of 2
#>   .. ..$ : NULL
#>   .. ..$ : chr [1:3] "t" "x" "y"
#>   [list output truncated]
#>  - attr(*, "class")= chr "tracks"
str( Neutrophils, list.len = 2 )
#> List of 411
#>  $ 21     : num [1:7, 1:3] 48 72 96 120 144 ...
#>   ..- attr(*, "dimnames")=List of 2
#>   .. ..$ : NULL
#>   .. ..$ : chr [1:3] "t" "x" "y"
#>  $ 22     : num [1:8, 1:3] 48 72 96 120 144 ...
#>   ..- attr(*, "dimnames")=List of 2
#>   .. ..$ : NULL
#>   .. ..$ : chr [1:3] "t" "x" "y"
#>   [list output truncated]
#>  - attr(*, "class")= chr "tracks"
```

Since there are quite many cells, we’ll sample just some of the tracks for the legibility of the plots in this tutorial – but everything we will do could also be done on the complete datasets.

```
# Take a sample
set.seed(1234)
TCells <- TCells[ sample( names(TCells), 30 ) ]
BCells <- BCells[ sample( names(BCells), 30 ) ]
Neutrophils <- Neutrophils[ sample( names(Neutrophils), 30 ) ]
```

Combine them in a single dataset, where labels also indicate celltype:

```
T2 <- TCells
names(T2) <- paste0( "T", names(T2) )
tlab <- rep( "T", length(T2) )

B2 <- BCells
names(B2) <- paste0( "B", names(B2) )
blab <- rep( "B", length(B2) )

N2 <- Neutrophils
names(N2) <- paste0( "N", names(Neutrophils) )
nlab <- rep( "N", length( N2) )

all.tracks <- c( T2, B2, N2 )
real.celltype <- c( tlab, blab, nlab )
```

# 1 Extracting a feature matrix

Using the function `getFeatureMatrix()`, we can quickly apply several quantification measures to all data at once (see `?TrackMeasures` for an overview of measures we can compute):

```
m <- getFeatureMatrix( all.tracks, 
                       c(speed, meanTurningAngle, 
                         outreachRatio, squareDisplacement) )

# We get a matrix with a row per track and one column for each metric:
head(m)
#>             [,1]      [,2]      [,3]      [,4]
#> T38   0.12164986 0.6895097 0.5000639 1271.0287
#> T115  0.12411080 0.8124751 0.4840314 3161.6797
#> T7954 0.13206831 1.5715280 0.3300338  109.4300
#> T5695 0.22796888 1.1175668 0.2823076  458.9368
#> T6386 0.07619656 1.8086072 0.4217940  137.8384
#> T7581 0.14522408 1.1691051 0.5149288 1558.9763
```

We can use this matrix to explore relationships between different metrics. For example, we can check the relationship between speed and mean turning angle:

```
plot( m, xlab = "speed", ylab = "mean turning angle" )
```

# 2 Dimensionality reduction methods: PCA, MDS, and UMAP

When using more than two metrics at once to quantify track properties, it becomes hard to visualize which tracks are similar to each other. Like with single-cell data, dimensionality reduction methods can help visualize high-dimensional track feature datasets. The function `trackFeatureMap()` is a wrapper method that helps to quickly visualize data using three popular methods: principal component analysis (PCA), multidimensional scaling (MDS), and uniform manifold approximate and projection (UMAP). The function `trackFeatureMap()` can be used for a quick visualization of data, or return the coordinates in the new axis system if the argument `return.mapping=TRUE`.

### 1.1 PCA

Use `trackFeatureMap()` to perform a principal component analysis (PCA) based on the measures “speed”, “meanTurningAngle”, “squareDisplacement”, “maxDisplacement”, and “outreachRatio” using the optional `labels` argument to color points by their real celltype and `return.mapping=TRUE` to also return the data rather than just the plot:

```
pca <- trackFeatureMap( all.tracks, 
               c(speed,meanTurningAngle,squareDisplacement,
                 maxDisplacement,outreachRatio ), method = "PCA", 
               labels = real.celltype, return.mapping = TRUE )
```

Note that the B cells and Neutrophils are relatively well-separated from each other in this plot, but the T cells are hard to distinguish from neutrophils based on these features.

We can then inspect the data stored in `pca`. This reveals, for example, that the first principal component is correlated with speed:

```
pc1 <- pca[,1]
pc2 <- pca[,2]
track.speed <- sapply( all.tracks, speed )
cor.test( pc1, track.speed )
#> 
#>  Pearson's product-moment correlation
#> 
#> data:  pc1 and track.speed
#> t = 4.5449, df = 88, p-value = 1.743e-05
#> alternative hypothesis: true correlation is not equal to 0
#> 95 percent confidence interval:
#>  0.2516387 0.5898408
#> sample estimates:
#>       cor 
#> 0.4360085
```

See `?prcomp` for details on how principal components are computed, and for further arguments that can be passed on to `trackFeatureMap()`.

### 1.2 MDS

Another popular method for visualization is multidimensional scaling (MDS), which is also supported by `trackFeatureMap()`:

```
trackFeatureMap( all.tracks,
               c(speed,meanTurningAngle,squareDisplacement,maxDisplacement,
                 outreachRatio ), method = "MDS",
               labels = real.celltype )
```

Internally, `trackFeatureMap()` computes a distance matrix using `dist()` and then applies MDS using `cmdscale()`. See the documentation of `cmdscale` for details and further arguments that can be passed on via `trackFeatureMap()`.

Again, we find that the B cells and Neutrophils are separated in this plot, while T cells mix with neutrophils.

### 1.3 UMAP

Uniform manifold approximate and projection (UMAP) is another powerful method to explore structure in high-dimensional datasets. `trackFeatureMap()` supports visualization of tracks in a UMAP. Note that this option requires the `uwot` package. Please install this package first using `install.packages("uwot")`.

```
trackFeatureMap( all.tracks,
        c(speed,meanTurningAngle,squareDisplacement,
          maxDisplacement,outreachRatio ), method = "UMAP",
          labels = real.celltype )
```

Also this plot confirms that B cells and neutrophils can be separated easily, but that T cells are somewhere in between.

# 3 Clustering: hierarchical clustering and k-means

To go beyond visualizing similar and dissimilar tracks using multiple track features, `clusterTracks()` supports the explicit grouping of tracks into clusters using two common methods: hierarchical and k-means clustering.

### 3.1 Hierarchical clustering

Hierarchical clustering is performed by calling `hclust()` on a distance matrix computed via `dist()` on the feature matrix:

```
clusterTracks( all.tracks,
               c(speed,meanTurningAngle,squareDisplacement,maxDisplacement,
                 outreachRatio ), method = "hclust", labels = real.celltype )
```

See methods `dist()` and `hclust()` for details.

### 3.2 K-means clustering

Secondly, `clusterTracks()` also supports k-means clustering of tracks. Note that this requires an extra argument `centers` that is passed on to the `kmeans()` function and specifies the number of clusters to make. In this case, let’s use three clusters because we have three celltypes:

```
clusterTracks( all.tracks,
               c(speed,meanTurningAngle,squareDisplacement,maxDisplacement,
                 outreachRatio ), method = "kmeans", 
               labels = real.celltype, centers = 3 )
```

In these plots, we see the value of each feature in the feature matrix plotted for the different clusters, whereas the color indicates the “real” celltype the track came from.
