# Supplementary material for: CelltrackR: An R package for fast and flexible analysis of immune cell migration data
Source: Immunoinformatics (Amst). Author manuscript; Available in PMC 2023 Apr 6. (PMC10079262; doi:10.1016/j.immuno.2021.100003)
Supplement: Supplemental movies and figures [file NIHMS1884788-supplement-Supplemental_movies_and_figures.zip › SF6-simulation.html]

Simulating Tracks


# Simulating Tracks

#### Inge Wortel

#### 2021-07-07

# Introduction

Simulating artifical tracks using mathematical or statistical models can help interpret the data from a tracking experiment. This tutorial will explain how to use simulation methods provided with the package. The simulation methods provided in the package are:

- `brownianTrack()` : a random walk in n dimensions.
- `beaucheminTrack()` : a special version of a random walk model specifically designed to describe T cell motion in an uninflamed lymph node.
- `bootstrapTrack()` : a data-driven simulation, where step speeds and turning angles are sampled from those observed in experimental data of interest.

# Datasets

First load the package:

```
library( celltrackR )
library( ggplot2 )
```

The package contains a dataset of T cells imaged in a mouse cervical lymph node using two-photon microscopy. We will here use this dataset as an example of how we can compare real tracking data with data from a simulation model. While the original data contained 3D coordinates, we’ll use the 2D projection on the XY plane (see the vignette on preprocessing of the package datasets for details).

The dataset consists of 199 tracks of individual cells in a tracks object:

```
str( TCells, list.len = 3 )
```

```
## List of 199
##  $ 1     : num [1:39, 1:3] 48 72 96 120 144 168 192 216 240 264 ...
##   ..- attr(*, "dimnames")=List of 2
##   .. ..$ : NULL
##   .. ..$ : chr [1:3] "t" "x" "y"
##  $ 3     : num [1:19, 1:3] 24 48 72 96 120 144 168 192 216 240 ...
##   ..- attr(*, "dimnames")=List of 2
##   .. ..$ : NULL
##   .. ..$ : chr [1:3] "t" "x" "y"
##  $ 4     : num [1:9, 1:3] 24 48 72 96 120 ...
##   ..- attr(*, "dimnames")=List of 2
##   .. ..$ : NULL
##   .. ..$ : chr [1:3] "t" "x" "y"
##   [list output truncated]
##  - attr(*, "class")= chr "tracks"
```

Each element in this list is a track from a single cell, consisting of a matrix with \((x,y)\) coordinates and the corresponding measurement timepoints:

```
head( TCells[[1]] )
```

```
##        t       x       y
## [1,]  48 90.8534 65.3943
## [2,]  72 89.5923 64.9042
## [3,]  96 88.6958 67.1125
## [4,] 120 87.3437 68.2392
## [5,] 144 86.2740 67.9236
## [6,] 168 84.0549 68.2502
```

# 1 Modelling brownian motion

### 1.1 A simple random walk

We can use the function `brownianTrack()` to simulate a simple random walk (here in 3 dimensions):

```
brownian <- brownianTrack( nsteps = 20, dim = 3 )
str(brownian)
```

```
##  num [1:21, 1:4] 0 1 2 3 4 5 6 7 8 9 ...
##  - attr(*, "dimnames")=List of 2
##   ..$ : NULL
##   ..$ : chr [1:4] "t" "x" "y" "z"
```

Note that this returns a single track matrix, which we can turn into a track object by using `wrapTrack()`:

```
plot( wrapTrack(brownian) )
```

We can also simulate multiple tracks at once using the `simulateTracks()` function. Do this and compare to the T cell tracks:

```
brownian.tracks <- simulateTracks( 10, brownianTrack( nsteps = 20, dim = 3 ) )
par(mfrow=c(1,2))
plot( brownian.tracks, main = "simulated random walk" )
plot( normalizeTracks( TCells ), main = "real T cell data" )
```

### 1.2 Matching displacement to data

In this simulation, the displacement vector for each step is sampled randomly in each dimension from a normal distribution of \(\mu=0, \sigma=1\). To match the average displacement observed in the T cell data:

```
# get displacement vectors
step.displacements <- t( sapply( subtracks(TCells,1), displacementVector ) )

# get mean and sd of displacement in each dimension
step.means <- apply( step.displacements, 2, mean )
step.sd <- apply( step.displacements, 2, sd )

# simulate brownian motion with the same statistics
brownian.tracks.matched <- simulateTracks( 10, brownianTrack( nsteps = 20, dim = 3,
                                                              mean = step.means,
                                                              sd = step.sd ) )

# compare displacement distributions
data.displacement <- sapply( subtracks( TCells,1), displacement )
matched.displacement <- sapply( subtracks( brownian.tracks.matched, 1 ), displacement )

df <- data.frame( disp = data.displacement,
                  data = "TCells" )
df2 <- data.frame( disp = matched.displacement,
                   data = "model" )
df <- rbind( df, df2 )
ggplot( df, aes( x = data, y = disp ) ) +
  geom_boxplot() +
  theme_classic()
```

```
# Plot new simulation versus real data
par(mfrow=c(1,2))
plot( brownian.tracks.matched, main = "simulated random walk" )
plot( normalizeTracks( TCells ), main = "real T cell data" )
```

> Note: this is not the best method for matching a Brownian model to data; the best method would be to use the diffusion coefficient as estimated using Furth’s equation. An example of this method is given later in this vignette.

### 1.3 A biased random walk

We can bias movement in a certain direction by setting the mean in each dimension:

```
# simulate brownian motion with bias
brownian.tracks.bias <- simulateTracks( 10, brownianTrack( nsteps = 20, dim = 3,
                                                              mean = c(1,1,1) ) )

plot( brownian.tracks.bias, main = "biased random walk" )
```

# 2 The Beauchemin model of lymphocyte migration

Aside from a simple random walk, the package also implements a slightly different model proposed by Beauchemin et al (2007). In contrast to the simple random walk, this model has a tuneable amount of persistence, and the cell pauses briefly between each step as it needs to reorient itself, which takes some time. For these reasons, the Beauchemin model generates tracks that are a bit more realistic than a simple random walk.

```
beauchemin.tracks <- simulateTracks( 10, beaucheminTrack(sim.time=20) )
plot( beauchemin.tracks )
```

See `?beaucheminTrack` for further details.

# 3 Bootstrapping method for simulating migration

The final simulation method provided in the package is `bootstrapTrack()`, which does not assume an underlying migration model but samples speeds and turning angles from a real dataset.

For example, we can do this for the T cell data:

```
bootstrap.tracks <- simulateTracks( 10, bootstrapTrack( nsteps = 20, TCells ) )
plot( bootstrap.tracks )
```

If all is as should be, the distribution of speed and turning angles should now match the real data closely. Check this:

```
# Simulate more tracks to reduce noice
bootstrap.tracks <- simulateTracks( 50, bootstrapTrack( nsteps = 20, TCells ) )

# Compare step speeds in real data to those in bootstrap data
real.speeds <- sapply( subtracks( TCells,1 ), speed )
bootstrap.speeds <- sapply( subtracks( bootstrap.tracks,1), speed )
dspeed <- data.frame( tracks = c( rep( "data", length( real.speeds ) ),
                                  rep( "bootstrap", length( bootstrap.speeds ) ) ),
                      speed = c( real.speeds, bootstrap.speeds ) )

# Same for turning angles
real.angles <- sapply( subtracks( TCells,2 ), overallAngle, degrees = TRUE )
bootstrap.angles <- sapply( subtracks( bootstrap.tracks,2), overallAngle, degrees = TRUE )
dangle <- data.frame( tracks = c( rep( "data", length( real.angles ) ),
                                  rep( "bootstrap", length( bootstrap.angles ) ) ),
                      angle = c( real.angles, bootstrap.angles ) )

# plot
pspeed <- ggplot( dspeed, aes( x = tracks, y = speed ) ) +
  geom_violin( color = NA, fill = "gray" ) +
  geom_boxplot( width = 0.3 ) +
  theme_classic()

pangle <- ggplot( dangle, aes( x = tracks, y = angle ) ) +
  geom_violin( color = NA, fill = "gray" ) +
  geom_boxplot( width = 0.3 ) +
  theme_classic()

gridExtra::grid.arrange( pspeed, pangle, ncol = 2 )
```

# 4 Example: Comparing data with models

### 4.1 Mean square displacement plot

To compare two different models with the real T cell data, we make a mean square displacement plot. To remove the effect of noise, we simulate a greater number of tracks than before:

```
# Simulate more tracks
brownian.tracks <- simulateTracks( 50, brownianTrack( nsteps = 20, dim = 3,
                                                              mean = step.means,
                                                              sd = step.sd ) )
bootstrap.tracks <- simulateTracks( 50, bootstrapTrack( nsteps = 20, TCells ) )

msd.data <- aggregate( TCells, squareDisplacement, FUN = "mean.se" )
msd.data$data <- "data"
msd.brownian <- aggregate( brownian.tracks, squareDisplacement, FUN = "mean.se" )
msd.brownian$data <- "brownian"
msd.bootstrap <- aggregate( bootstrap.tracks, squareDisplacement, FUN = "mean.se" )
msd.bootstrap$data <-"bootstrap"

msd <- rbind( msd.data, msd.brownian, msd.bootstrap )
ggplot( msd, aes( x = i, y = mean, ymin = lower, ymax = upper, color = data, fill = data ) ) +
  geom_ribbon( color= NA, alpha  = 0.2 ) +
  geom_line() +
  labs( x = "t (steps)",
        y = "square displacement" ) +
  scale_x_log10(limits= c(NA,10) ) +
  scale_y_log10() +
  theme_bw()
```

```
## Warning: Removed 49 row(s) containing missing values (geom_path).
```

Both the random walk model and the bootstrapped tracks underestimate the MSD as observed in real data – even though the bootstrapped data at least matches speed and turning angle distributions well.

This suggests there is more directional persistence in the real data than those models assume. In the next section, we will check this by making the autocorrelation plot.

### 4.2 Persistence: autocovariance plot

To check for directional persistence, we generate an autocovariance plot:

```
# compute autocorrelation
acor.data <- aggregate( TCells, overallDot, FUN = "mean.se" )
acor.data$data <- "data"
acor.brownian <- aggregate( brownian.tracks, overallDot, FUN = "mean.se" )
acor.brownian$data <- "brownian"
acor.bootstrap <- aggregate( bootstrap.tracks, overallDot, FUN = "mean.se" )
acor.bootstrap$data <-"bootstrap"

acor <- rbind( acor.data, acor.brownian, acor.bootstrap )
ggplot( acor, aes( x = i, y = mean, ymin = lower, ymax = upper, color = data, fill = data ) ) +
  geom_ribbon( color= NA, alpha  = 0.2 ) +
  geom_line() +
  labs( x = "dt (steps)",
        y = "autocovariance" ) +
  scale_x_continuous(limits= c(0,10) ) +
  theme_bw()
```

```
## Warning: Removed 49 row(s) containing missing values (geom_path).
```

Indeed, the autocovariance drops less steeply for the real T cell data, which indicates that there is a directional persistence in the T cell data that is not captured by the brownian and bootstrapping models. Thus, even when a model captures some aspects of the walk statistics in the data, it may still behave differently in other respects.

# 5 Fitting models on the MSD

In the above, we saw that a Brownian motion model underestimated the MSD of the real T-cell dataset. Here, we show how we can do better by fitting models on the MSD.

### 5.1 Before we start: dimensionality and imaging windows

Before we start, let’s look at the data we are fitting in more detail. If we look at the dimensions of the T-cell dataset:

```
boundingBox( TCells )
```

```
##       t     x       y
## min  24   0.4   0.400
## max 960 409.2 334.724
```

we see that the imaging window is limited to roughly 200 x 200 x 50 \(\mu\)m; thus, it is smaller in the z-dimension.

A limited imaging window like this can cause artefacts in the MSD curve because cells can not be followed after they leave the imaging window. Thus, for larger \(\Delta t\), we underestimate the mean (squared) displacement because the faster cells have left the imaging window and do not end up in our data. In our case, this will be particularly problematic in the z-dimension. Thus, for now we will not fit the 3D data, but instead use the 2D projection on the xy-plane:

```
Txy <- projectDimensions( TCells, c("x","y") )
```

Let’s look at the MSD curve we get:

```
# Compute MSD
msdData <- aggregate( Txy, squareDisplacement, FUN = "mean.se" )

# Scale time axis: by default, this is in number of steps; convert to minutes.
tau <- timeStep( Txy ) / 60 # in minutes
msdData$dt <- msdData$i * tau

# plot
ggplot( msdData, aes( x = dt, y = mean ) ) +
  geom_ribbon( alpha = 0.3, aes( ymin = lower, ymax = upper ) ) +
  geom_line() +
  labs( x = expression( Delta*"t (min)" ), 
        y = expression( "displacement"^2*"("*mu*"m"^2*")") ) +
  coord_cartesian( xlim = c(0,NA), ylim = c(0,NA), expand = FALSE ) +
  theme_bw()
```

Two things are worth noting here:

1. the larger \(\Delta\)t, the higher the uncertainty (SE); this is because at larger \(\Delta\)t, we have fewer subtracks of that length, and thus less data to compute a mean on;
2. the MSD curve has a weird shape. This is actually the case because of the limited imaging window problem described above. We will therefore use only the first part of the curve (the first 5 minutes), where we have enough data and the imaging window problem remains manageable.

```
fitThreshold <- 5 # minutes
```

Now, let’s see how we can fit the two simulation models: `brownianTrack` and `beaucheminTrack` on this MSD curve.

### 5.2 Fitting Brownian motion based on the diffusion coefficient

The `brownianTrack` function takes 4 arguments: the number of steps to simulate, the number of dimensions to simulate in, and the mean and sd of the step sizes $(x, y, z) in each dimension. The number of steps depends on the time we wish to simulate, the dimension is 2 (since we projected the T cells on the xy plane) and the mean of an unbiased random walk is always 0. That leaves the standard deviation.

For Brownian motion, the dimension-wise variance of the step size is related to the “diffusion coefficient” \(D\) as:

\[\text{var} = 2D\tau\] with \(\tau\) the step duration. For the standard deviation, we get: \[\sigma\_\text{dim} = \sqrt{2D\tau}\] we already know the \(\tau\) of our data, and can obtain \(D\) by fitting Furth’s formula for the mean squared displacement \(\langle d^2 \rangle\):

\[\langle d^2 \rangle (\Delta t) = 2Dn\_d \big( \Delta t - P(1-e^{-\Delta t/P} ) )\] Here, \(D\) is the diffusion coefficient, \(P\) the persistence time, \(\Delta t\) the time resolution we are looking at, and \(n\_d\) the number of dimensions.

We can fit this function using R’s `nls()` on the MSD data to get \(D\) and \(P\):

```
fuerthMSD <- function( dt, D, P, dim ){
  return( 2*dim*D*( dt - P*(1-exp(-dt/P) ) ) )
}

# Fit this function using nls. We fit only on the data where 
# dt < fitThreshold (see above), and need to provide reasonable starting
# values or the fitting algorithm will not work properly. 
model <- nls( mean ~ fuerthMSD( dt, D, P, dim = 2 ), 
              data = msdData[ msdData$dt < fitThreshold, ], 
              start = list( D = 10, P = 0.5 ), 
              lower = list( D = 0.001, P = 0.001 ), 
              algorithm = "port" 
)
D <- coefficients(model)[["D"]] # this is now in units of um^2/min
P <- coefficients(model)[["P"]] # persistence time in minutes
D
```

```
## [1] 19.91724
```

```
P
```

```
## [1] 0.3069776
```

Note that we will not use \(P\). However, we fit Furth’s formula with a non-zero persistence time \(P\) because we would otherwise underestimate \(D\).

Now, given \(D\), simulate some tracks:

```
# simulate tracks using the estimated D. We simulate with time unit seconds
# to match the data, so divide D by 60 to go from um^2/min to um^2/sec.
# The dimension-wise variance of brownian motion is 2*D*tau (with tau the step
# duration), so use this to parametrise the model:
tau <- timeStep( Txy )
brownianScaled <- function( nsteps, D, tau, dim = 2 ){
  tr <- brownianTrack( nsteps = nsteps, dim = 2, sd = sqrt( 2*(D/60)*tau ) )
  tr[,"t"] <- tr[,"t"]*tau # scale time
  return(tr)
}
brownian.tracks <- simulateTracks( 1000,  
                                   brownianScaled( nsteps = maxTrackLength(Txy ),
                                                   D = D, tau = tau ) )
plot( brownian.tracks[1:10], main = "brownian motion")
```

Finally, get MSD and compare it to data:

```
# get msd of the simulated tracks and scale time to minutes
msdBrownian <- aggregate( brownian.tracks, squareDisplacement, FUN = "mean.se" )
tau <- timeStep( brownian.tracks ) / 60 # in minutes
msdBrownian$dt <- msdBrownian$i * tau

# Get Furth MSD using fitted P and D for comparison
msdFuerth <- data.frame( dt = msdBrownian$dt, 
                         mean = fuerthMSD( msdBrownian$dt, D, P, dim = 2 ))

# plot
ggplot( msdBrownian, aes( x = dt, y = mean) ) +
  geom_point( data = msdData, shape = 21, aes( fill = dt < fitThreshold ), color = "blue", show.legend = FALSE ) +
  geom_line( data = msdFuerth, color = "red", lty = 2 ) +
  geom_line( )+
  scale_fill_manual( values = c( "TRUE" = "blue", "FALSE" = "transparent" ) ) +
  scale_x_log10( expand = c(0,0) ) +
  scale_y_log10( expand = c(0,0) ) +
  labs( color = NULL, x = expression( Delta*"t (min)"), 
        y = expression( "displacement"^2*"("*mu*"m"^2*")") , fill = NULL ) +
  theme_bw()
```

Here, we see the data in blue, where the colored circles are the data the fit is based on. The red line is the Furth’s MSD, the black line is the Brownian model. The Brownian model does not describe the data well especially on short time scales, because it does not contain the persistence that the Furth formula does. Nevertheless, this fit is better than the one with matched displacements on short time scales.

Next, let’s look at a slightly more complicated model.

### 5.3 Fitting the Beauchemin model

We now use a similar approach to fit the model proposed by Beauchemin et al (2007), which was designed for T-cell migration in the lymph node.

The MSD of this model can be derived analytically (Textor et al (2013), proposition 12) :

\[\langle d^2 \rangle (\Delta t)= 2Mt - 2Mt\_\text{free} \cdot \begin{cases}
\frac{1}{3} & t \geq t\_\text{free} \\
\frac{1}{3} \big(t/t\_\text{free}\big)^3 - \big(t/t\_\text{free}\big)^2 + \big(t/t\_\text{free}\big) & t < t\_\text{free}
\end{cases}\]

where \(M\) is the motility coefficient, which should be the same as our diffusion coefficient \(D\) (see above). \(M\) is related to the model parameters (Textor et al (2013), equation 2):

\[M = \frac{v\_\text{free}^2\cdot t\_\text{free}^2}{6(t\_\text{free}+t\_\text{pause})}\]

We should note here that the Beauchemin has three parameters (\(v\_\text{free}\), \(t\_\text{free}\), \(t\_\text{pause}\)), but that these are underdefined based on MSD: multiple parameter combinations can give the exact same MSD (see Beauchemin et al (2007), Textor et al (2013)). This means we cannot fit all three parameters based on the MSD. Here, we’ll set \(t\_\text{pause}\) to the literature value of 0.5 minute, and fit the other parameters \(v\_\text{free}\) and \(t\_\text{free}\) based on the MSD to show how this works:

```
# analytical formula
beauchemin.msd <- function( x, M=60, t.free=2, t.pause=0.5, dim=2 ){
  v.free <- sqrt( 6 * M * (t.free+t.pause) ) / t.free
  M <- (v.free^2 * t.free^2) / 6 / (t.free + t.pause)
  multiplier <- rep(1/3, length(x))
  xg <- x[x<t.free]
  multiplier[x<t.free] <- 1/3 * (xg/t.free)^3 - (xg/t.free)^2 + (xg/t.free)
  2 * M * x * dim - 2 * M * dim * t.free * multiplier
}
# Again, fit on the first fitThreshold min, before confinement becomes an issue.
beaucheminFit <- nls( mean ~ beauchemin.msd( dt, M, t.free ), 
                      msdData[ msdData$dt < fitThreshold, ], 
                      start=list(M=30, t.free=1))

# extract parameters M and t.free
M <- coef(beaucheminFit)[["M"]] # in um^2/min
t.free <- coef(beaucheminFit)[["t.free"]] # in min
```

If everything went correctly, \(M\) should not be too different from the diffusion coefficient \(D\) we fitted earlier:

```
c( D = D, M = M )
```

```
##        D        M 
## 19.91724 19.84664
```

Indeed, they are very similar, which is good news.

Using these fitted values, get the MSD and compare to data:

```
# Get the fitted MSD using these values
msdBeauchemin <- data.frame(
  dt = msdBrownian$dt,
  mean = beauchemin.msd( msdBrownian$dt, M, t.free )
)

# plot
ggplot( msdBeauchemin, aes( x = dt, y = mean) ) +
  geom_point( data = msdData, shape = 21, aes( fill = dt < fitThreshold ), color = "blue", show.legend = FALSE ) +
  geom_line( )+
  scale_fill_manual( values = c( "TRUE" = "blue", "FALSE" = "transparent" ) ) +
  scale_x_log10( expand = c(0,0) ) +
  scale_y_log10( expand = c(0,0) ) +
  labs( color = NULL, x = expression( Delta*"t (min)"), 
        y = expression( "displacement"^2*"("*mu*"m"^2*")") , fill = NULL ) +
  theme_bw()
```

This model seems to fit the data much better!

Now, let’s use the discovered parameters to simulate some tracks on a longer time scale:

```
# t.free has been fitted; set t.pause to its fixed value and 
# compute v.free from the fitted M:
t.pause <- 0.5 # fixed
v.free <- sqrt( 6 * M * (t.free+t.pause) ) / t.free

# simulate 3 tracks using the fitted parameters.
# we don't use the arguments p.persist, p.bias, bias.dir, and taxis.mode,
# since these are not parameters of the original Beauchemin model.
tau <- timeStep( Txy )
beauchemin.tracks <- simulateTracks( 3,  
                                   beaucheminTrack( sim.time = 10*max( timePoints( Txy) ),
                                                    delta.t = tau,
                                                    t.free = t.free,
                                                    v.free = v.free,
                                                    t.pause = t.pause ) )

plot( beauchemin.tracks, main = "Beauchemin tracks")
```
