# Supplementary material for: CelltrackR: An R package for fast and flexible analysis of immune cell migration data
Source: Immunoinformatics (Amst). Author manuscript; Available in PMC 2023 Apr 6. (PMC10079262; doi:10.1016/j.immuno.2021.100003)
Supplement: Supplemental movies and figures [file NIHMS1884788-supplement-Supplemental_movies_and_figures.zip › SF7-cheatsheet.pdf.pdf]

Analyzing cell migration data in R

CelltrackR cheat sheet

To analyze cell movement, we record a cell's coordinates in time-lapse videos to obtain a cell *track*. To facilitate the interpretation of tracking data, **celltrackR** implements a large variety of methods for the fast and flexible analysis of track data in R. Load data from a text file, get rid of artefacts and tracking errors by performing quality controls proposed in literature, and analyze any metric on the level of individual tracks, steps, or subtracks. **CelltrackR** supports angle analyses and allows rapid visualization, clustering, and simulation of tracks. Let's get started!

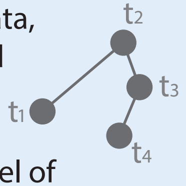

3. Subsetting data: single tracks, (staggered) subtracks, and steps

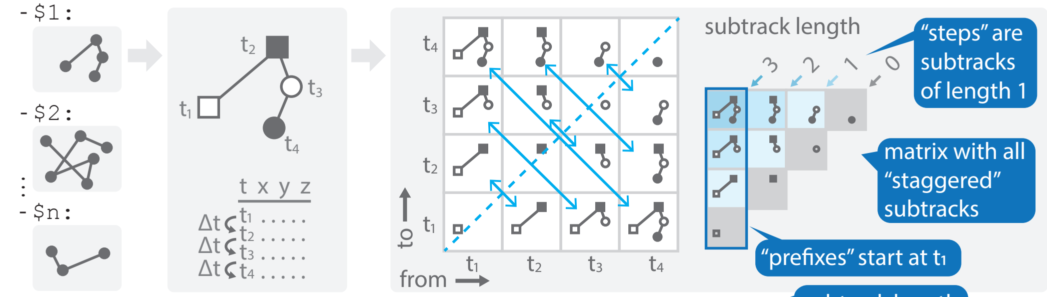

use [[ ]] to return coordinate matrix, [ ] to return a tracks object.

```
cell.steps <- subtracks( x, 1 )
all.steps <- subtracks( X, 1 )
first.steps <- prefixes( X, 1 )
t.steps <- subtracksByTime( X, t, 1 )
```

subtrack length

all subtracks starting at t

1. Loading & converting tracks

Generate tracks object from a csv file:

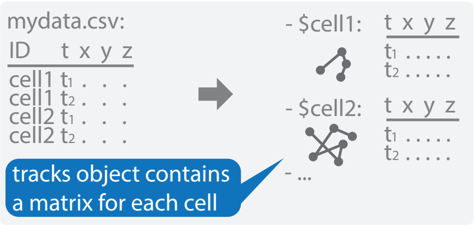


```
read.tracks.csv( mydata.csv,
 id.column = 1, time.column = 2,
 pos.columns = 3:5 )
```

Concatenate two tracks objects:

```
c( X1, X2 )
```

Convert between data structures:

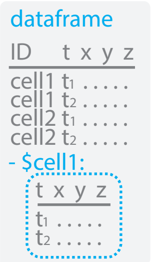

`as.data.frame( X )` tracks to dataframe

`as.tracks( D )` dataframe to tracks

`as.list( X )` tracks to regular R list

`wrapTrack( x )` wrap single track matrix into a track object

Sort tracks by time-order:

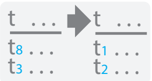


```
sort( X )
```

Output of `read.tracks.csv()` and `as.tracks.data.frame()` is time-ordered by default.

2. Quality control & preprocessing

Longer tracks allow better inference of the cell's behavior, especially in cell-based analyses (**box 4**).

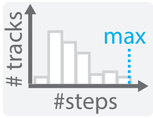

`hist( sapply( X, nrow ) )` length distribution

`maxTrackLength( X )` longest track (# steps)

function must return `TRUE/FALSE`

```
filterTracks( function(x) nrow(x)>n, X )
```

keep only tracks of at least n steps

Filtering can cause bias. Consider a step-based analysis (**box 4**) instead of removing short tracks.

Check for **unequal Δt** between steps, or gaps:

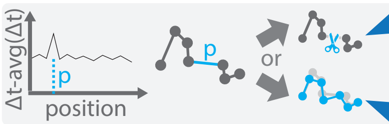

split into two tracks

interpolate @fixed Δt

```
avdt <- timeStep( x ); hist( sapply(
  subtracks( x, 1 ), duration ) - avdt )
```

Fix this issue automatically for all tracks in X with an irregular Δt above some threshold:

or: "split"

```
fix1 <- repairGaps( X, "interpolate" )
```

Adjust **time resolution Δt**:

subsample every k-th timepoint

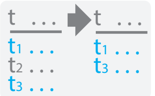


```
subsample( x, k = 2 )
interpolateTrack( x, dtvec )
```

interpolate at times in dtvec

Angle analyses (**box 6**) can help detect artifacts, drift, and tracking errors (**Beltman et al, 2009**).

4. Analysis types: cell-based, step-based, and staggered metrics

Track properties can be computed in a cell-based, step-based, or staggered fashion. For more information, please refer to (**Beltman et al, 2009**). Examples are shown for the analysis of speed, but can also be performed with other analysis measures (**box 5**).

Cell-based

Find average speed of each individual cell (track):

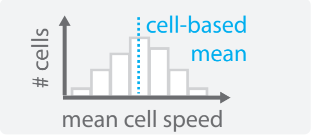


```
mean( sapply( X, speed ) )
```

cells have equal weights; *steps* from short tracks weigh more

Get instantaneous/"step" speed distribution for each cell (track):

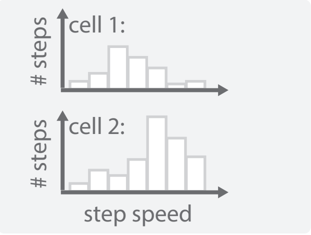


```
steps <- subtracks( x, 1 )
hist( sapply(
 steps, speed ) )
```

steps of one cell x

Step-based

Average speed over all steps, pooled from all tracks together:

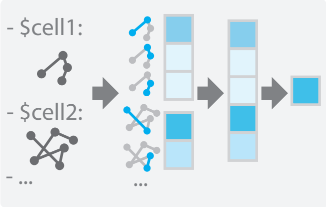


```
aggregate( X, speed,
 subtrack.length = 1,
 FUN = mean )$value
```

steps have equal weights; *cells* with longer tracks weigh more

To get the distribution over all steps instead of only the mean:

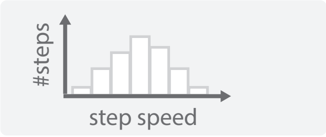


```
steps <- subtracks( X, 1 )
hist( sapply(
 steps, speed ) )
```

all steps in object X

Staggered

Measure speed on all subtracks in the staggered matrix:

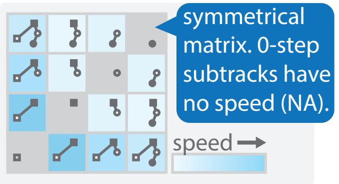

symmetrical matrix. 0-step subtracks have no speed (NA).

```
image( applyStaggered(
 x, speed, matrix = TRUE ) )
```

if `FALSE`: return only the matrix mean, which is dominated by short (more frequent!) subtracks.

Directly get all mean cell speeds (over the staggered subtracks):

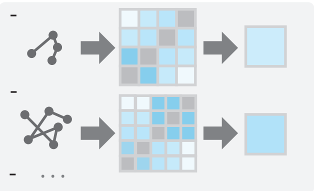


```
sapply( X,
 staggered( speed ) )
```

5. Analysis measures
(see also `?TrackMeasures`)

Speed and displacement

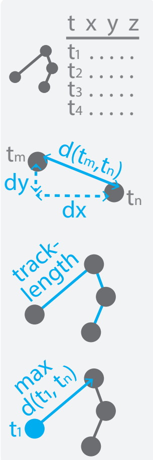

`duration( x )`
$= t_{end} - t_1$

`displacement( x, from = m, to = n )`
$= d(t_m,t_n)$

see also:
`squareDisplacement()`
`displacementVector()`
`normalizeToDuration()`

`trackLength( x )`
$= d(t_1,t_2) + \ldots + d(t_{end-1},t_{end})$

`speed( x )`
= tracklength/duration

`maxDisplacement( x )`
= max $d(t_1, t_n)$

Track straightness

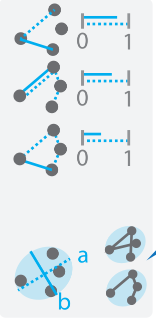

`displacementRatio( x )`
$= d(t_1,t_{end})$/max d

`outreachRatio( x )`
= max d/tracklength

`straightness( x )`
$= d(t_1,t_{end})$/tracklength

note that asphericity ignores time-ordering

`asphericity( x )`
$= (a^2 - b^2)^2/(a^2 + b^2)^2$

Turning angles

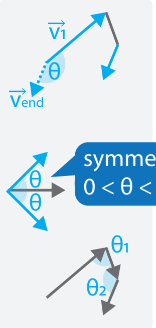

`overallAngle( x )`
= angle $\theta(\vec{v_1}, \vec{v_{end}})$ (first & last step)

`overallDot( x )`
= dot product $\vec{v_1} \cdot \vec{v_{end}} = \frac{\cos\theta}{|\vec{v_1}||\vec{v_{end}}|}$

symmetric $0 < \theta < \pi$

useful for autocorrelation/ autocovariance plots

`meanTurningAngle( x )`
$= \text{mean } (\theta_1, \ldots, \theta_{end})$

6. Angles & Directionality
(see also `?AngleAnalysis`)

Angles to a reference point, direction, or plane

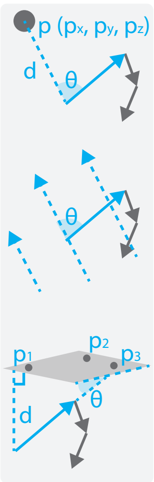

`angleToPoint( x,p )`
= angle θ between first step and reference point

`distanceToPoint( x,p )`
= distance d between first step and reference point

`angleToDir( x,dvec )`
= angle θ between first step and reference direction

`angleToPlane( x,p1,p2,p3 )`
= angle θ between first step and plane with points p1-p3

`distanceToPlane( x,p1,p2,p3 )`
= distance d between first step and plane with points p1-p3

Angles between pairs of steps or tracks can help identify directional biases or artefacts (**Beltman et al, 2009**):

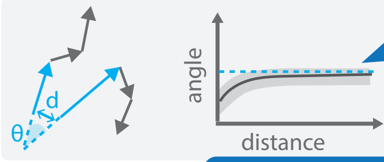

or try `analyzeCellPairs()`

```
step.pairs <- analyzeStepPairs( X )
plot( step.pairs$dist, step.pairs$angle )
```

Hotelling's test can help detect global directionality in a dataset in an unbiased fashion (**Textor et al, 2011**):

```
hotellingsTest( X,
  plot = TRUE )
```

does the average step displacement differ from the null vector?

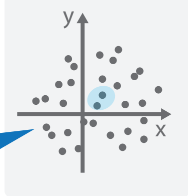

7. Visualization & Clustering:
detecting patterns in track data

Visualizing tracks in space

3D tracks? see `plot3d()` & `projectDimensions()`

```
plot( X )
plot(normalizeTracks( X ))
```

overlay track starting points

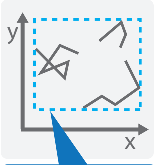

`boundingBox(X)`

Track measures by subtrack Δt: mean square displacement (MSD) & autocovariance plots

```
plot(aggregate( X, squareDisplacement ))
plot(aggregate( X, overallDot ))
```

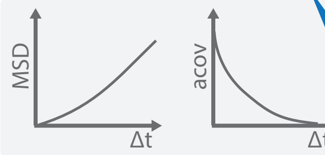

Plot `cos()` of `overallAngle()` for an autocorrelation plot

Tracks in feature space: Visualize two measures in a scatterplot:

```
plotTrackMeasures(
  X, speed,
  meanTurningAngle )
```

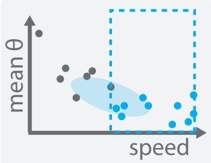

Or subset tracks by one feature first:

```
minv <- median(
  sapply( X, speed ) )
fast <- selectTracks(
  X, speed, minv, Inf )
```

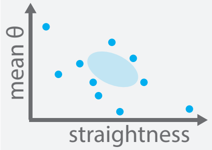

Or visualize higher dimensional feature sets with dimensionality reduction:

```
trackFeatureMap( X,
  c(speed,straightness,
  meanTurningAngle),
  method = "PCA" )
```

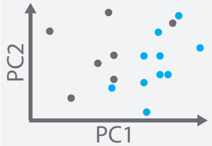

Other methods: `"UMAP"/"MDS"`

Cluster tracks by features:

```
clusterTracks( X,
  c(speed,straightness,
  meanTurningAngle),
  method = "hclust" )
```

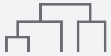

Or: `"kmeans"`

8. Simulating tracks:
Models & bootstrapping

Comparing observed data to idealized models is useful for interpretation. CelltrackR supports several methods for simulating tracks.

A **random walk** in dim dimensions:

```
brownianTrack( nsteps, dim, mean=c(0,0),
  sd=c(1,1) )
```

non-zero for directional bias

A **"stop-and-go"** model designed for T cells (**Beauchemin et al, 2007**). Cells move at speed v.free for time t.free, and then pause for a time t.pause before changing direction (can be with directional persistence or directional bias):

```
beaucheminTrack( sim.time, delta.t,
  p.persist, p.bias, bias.dir, taxis.mode,
  t.free, v.free, t.pause )
```

unlike `brownianTrack()`, `beaucheminTrack()` has an explicit definition of time.

A **bootstrapped track** matches speeds and turning angles to those observed in data:

```
bootstrapTrack( nsteps, X )
```

Simulate multiple tracks at once:

```
simdata <- simulateTracks( 10,
  bootstrapTrack( nsteps, X ) )
```

or another simulation method

References

Beauchemin et al (2007). Characterizing T cell movement within lymph nodes in the absence of antigen. *Journal of Immunology*.

Beltman et al (2009). Analysing Immune cell migration. *Nature Reviews Immunology*.

Mokhtari et al (2013). Automated characterization and parameter-free classification of cell tracks based on local migration behavior. *PLoS ONE*.

Textor et al (2011). Defining the quantitative limits of intravital two-photon lymphocyte tracking. *PNAS*.

Learn more?
Check out the detailed examples in the package vignettes:

```
browseVignettes( package = "celltrackR" )
```


© Johannes Textor, Katharina Dannenberg, Jeffrey Berry, Gerhard Burger, Inge Wortel (2019).

For the newest version, visit: https://github.com/ingewortel/celltrackR
To cite celltrackR, please refer to: `citation( "celltrackR" )`.
